# Targeted classification of metal–organic frameworks in the Cambridge structural database (CSD)†
†Electronic supplementary information (ESI) available: Details of protocols used to identify CSD MOF families (PDF), the corresponding Conquest queries to look for different MOF families and functional groups, details of MOFs geometrical properties calculations and crystal quality assessment (PDF), the bash script used for the quick identification of structures with missing hydrogens and occupancy issues, linked CSD refcodes for MOF families and dimensionalities (XLSX), animated GIFs for geometric properties of MOFs, Python script to determine framework dimensionality, GCMC simulation files and updates on the CSD MOF subset (PDF). See DOI: 10.1039/d0sc01297a


**DOI:** 10.1039/d0sc01297a

**Published:** 2020-06-17

**Authors:** Peyman Z. Moghadam, Aurelia Li, Xiao-Wei Liu, Rocio Bueno-Perez, Shu-Dong Wang, Seth B. Wiggin, Peter A. Wood, David Fairen-Jimenez

**Affiliations:** a Adsorption & Advanced Materials Laboratory (AAML) , Department of Chemical Engineering & Biotechnology , University of Cambridge , Philippa Fawcett Drive , Cambridge CB3 0AS , UK . Email: df334@cam.ac.uk; b Dalian National Laboratory for Clean Energy , Dalian Institute of Chemical Physics , Chinese Academy of Sciences , 457 Zhongshan Road , Dalian 116023 , P. R. China; c The Cambridge Crystallographic Data Centre , 12 Union Road , Cambridge , UK; d University of Chinese Academy of Sciences , 19A Yuquan Road , Beijing 100049 , P. R. China

## Abstract

Large-scale targeted exploration of metal–organic frameworks (MOFs) with characteristics such as specific surface chemistry or metal-cluster family has not been investigated so far. These definitions are particularly important because they can define the way MOFs interact with specific molecules (*e.g.* their hydrophilic/phobic character) or their physicochemical stability. We report here the development of algorithms to break down the overarching family of MOFs into a number of subgroups according to some of their key chemical and physical features. Available within the Cambridge Crystallographic Data Centre's (CCDC) software, we introduce new approaches to allow researchers to browse and efficiently look for targeted MOF families based on some of the most well-known secondary building units. We then classify them in terms of their crystalline properties: metal-cluster, network and pore dimensionality, surface chemistry (*i.e.* functional groups) and chirality. This dynamic database and family of algorithms allow experimentalists and computational users to benefit from the developed criteria to look for specific classes of MOFs but also enable users – and encourage them – to develop additional MOF queries based on desired chemistries. These tools are backed-up by an interactive web-based data explorer containing all the data obtained. We also demonstrate the usefulness of these tools with a high-throughput screening for hydrogen storage at room temperature. This toolbox, integrated in the CCDC software, will guide future exploration of MOFs and similar materials, as well as their design and development for an ever-increasing range of potential applications.

## Abstract 2

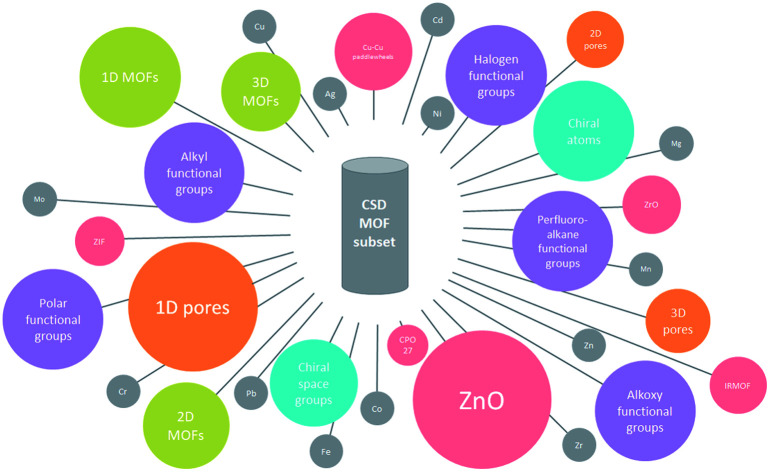
Large-scale targeted exploration of metal–organic frameworks (MOFs) with characteristics such as specific surface chemistry or metal-cluster family has not been investigated so far.

## 

Developed two decades ago, metal–organic frameworks (MOFs) have attracted an enormous attention in the field of porous materials.^1^–^7^ Owing to their chemical diversity and structural variety, MOFs have been intensely explored to target industrial challenges including gas storage^8^–^12^ and separation,^13^–^17^ catalysis,^18^–^20^ chemical sensing,^21^–^23^ biomedical imaging as well as biomolecule encapsulation and drug delivery.^24^–^29^ Because of their synthetic flexibility, the number of reported MOF materials has increased dramatically in the past decade.

Given their interest and the vast amount of research done in this area, the nature of MOFs has been under intense debate for some years, creating a philosophical debate that can be linked to Wittgenstein's *Tractatus*, *i.e.* the identification of the relationship between language and reality and the definition of the limits of science.^30^ At this point, several research groups have developed different MOF databases based on hypothetical or experimental materials in order to study the domains of applicability of MOFs.^31^–^34^ Mostly used for gas adsorption and separation, the experimental materials databases focused on porous MOFs present in the Cambridge Structural Database (CSD)^35^ at the time of their publication. This effort to compile MOF structures resulted in outstanding tools for their screening and study but had some issues related to their regular update. To solve this problem, we described in the past a complete collection of MOF materials in the CSD, providing users access to all the existing MOF materials through a single real-time updating resource. As of January 2020, a staggering 99 075 MOFs exist in the CSD MOF subset (2020.0 CSD release),^1^ fully integrated into ConQuest,^36^ the primary structural search software developed by the Cambridge Crystallographic Data Centre (CCDC).

MOF databases in conjunction with molecular simulations have proven to be extremely useful for the exploration of structure–property landscapes and screening of MOFs to find optimal materials. This can be exemplified by the efforts of the United States Materials Genome Initiative, aiming to accelerate the way materials are developed and deployed to market.^37^ In spite of the enormous advances implemented in high-throughput simulations (HTS) and data mining, no standard convention exists on how MOFs can be classified based on their important chemical and structural anatomy. Indeed, previous studies focused on the computational geometric analysis of structures such as surface area, pore size and void fraction. This is clearly useful for performing brute-force HTS for gas adsorption and/or separation in the entire structural phase space, giving a birds-eye point of view on property–performance relationships. Despite being of huge interest for experimentalists, large-scale targeted exploration of MOFs with specific characteristics such as a given chemical functionality, or a family of specific metal-cluster, has not been widely explored so far. A MOF identification scheme was recently developed to enable rapid data searches amongst the existing databases.^38^ The open software decomposes the structure and topology of a given MOF using standard cheminformatics formats to assign a unique identifier to the MOF. In this process, interesting information can be extracted from MOF databases, such as most common linkers, polymorphs and topologies. The necessity for such capabilities results from the MOF community's growing knowledge on the advantages and challenges of MOFs, which has enabled them to focus their research interests on certain chemistries deemed relevant to their practice – an excellent example is the recognition of the outstanding stability of Zr-MOFs. By breaking down the big family of MOFs into smaller hierarchical categories of materials that exhibit similar features, researchers would benefit from a clearer evaluation on how the MOF landscape is structured in terms of what materials have already been synthesized. Precise identification of different classes of materials, as opposed to brute-force screening, can also significantly improve the way they are studied for different applications.

As part of the CCDC's efforts to categorize crystalline materials, we report here the classification of MOFs according to some of their key features and their evolution over time since they were first synthesized. Although the methods presented here do not represent a standardized approach to the classification of MOFs, we believe these simple tools can help MOF researchers navigate through the data available and highlight the necessity to establish such standards. For easier data exploration, we compiled all the obtained information and built an interactive data visualization website at http://aam.ceb.cam.ac.uk/mof-explorer/CSD_MOF_subset.

## A CSD-integrated toolbox for the exploration of the CSD MOF subset

In our previous work, we released a set of scripts for the removal of bound and unbound solvents, useful for processing the structural data before further calculations. To enable easy data exploration of the CSD MOF subset, we present here two additions to the CSD toolbox consisting of: (i) ConQuest and CSD Python API search queries and methods for specific types of MOFs and (ii) a new script for the determination of framework dimensionality. This toolbox uses the CCDC software package and can therefore be applied to the CSD MOF subset directly. First, we categorize MOFs into some of the most well-known secondary building units (SBU) and functional groups, providing the possibility of looking for specific families of MOFs within the CSD using a combination of the CSD Python API and the *Draw* function in ConQuest. The latter enables users to define specific structural criteria corresponding to their target type of structures. We provide an example of methods used for such a targeted search later on in this paper. We also include here a specific group of chiral MOFs, identified with the CSD Python API. Second, we investigate the dimensionality of MOF networks using an in-house script. This algorithm generates the smallest box containing the smallest repeating unit of each structure. The latter is then expanded and a new smallest-containing box is created. The dimensions of the initial box and the last box are then compared to determine in which directions the structure has expanded. The script was tested on 1/5th of 52 787 structures (*i.e.* 11 515). The results were compared to those obtained with Zeo++,^39^ an open-source software that is able to determine framework dimensionality based on atom connectivity. 30% (*i.e.* 3663) of the results disagreed, which led to the visual inspection of 2157 of these structures. We found that our in-house script was correct in 93% of the cases where there was a disagreement. Based on these comparisons and checks, we estimated our predictions to be overall 97% accurate. The results obtained with these tools are presented later on in this paper, and further details of these tools are available in the ESI.† These new features – all integrated in the CSD – will allow users to have access to some of the most widely studied classes of MOFs in a single resource and offer a unique platform to boost the applicability of MOFs for a wide range of uses from gas storage/separation to asymmetric catalysis and enantiomer separation. Researchers can use the algorithms developed here to exploit the most recent MOF subset in the CSD release and maintained by the CCDC every quarter.^1^ The principles outlined here are also customizable if need be; therefore, we encourage users to develop similar algorithms for new families of MOFs according to their interests, where the structures can be downloaded for computational studies.

## Textural properties of MOFs and their evolution

**Fig. 1:**
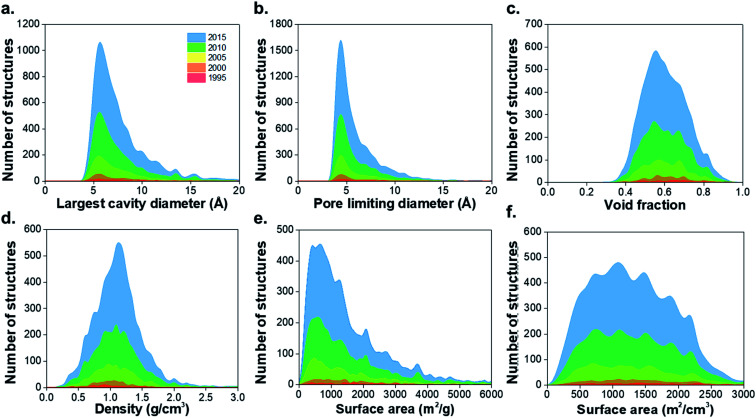
Histograms comparing geometric properties for all the porous MOFs in the CSD MOF subset from 1995 to 2015. (a) Largest cavity diameter (LCD), (b) pore limiting diameter (PLD), (c) void fraction, (d) density, (e) gravimetric surface area, (f) volumetric surface area. The animated version of these graphs can be found in the ESI.† All family-property relationships of the 8253 porous MOFs presented in this work can be found online at ; http://aam.ceb.cam.ac.uk/mof-explorer/CSD_MOF_subset.

The structural characterization discussed here is focused on the porous MOFs from the CSD MOF subset version 5.37.^1^ From a total of 55 547 non-disordered structures in the *non-disordered MOF subset*, we excluded a number of MOFs from the structural analysis due to presence of partial occupancy issues (583 MOFs) and those containing missing framework hydrogens (2177 MOFs), leaving 52 787 structures. 8253 materials were found to be porous according to previously described criteria, *i.e.* a nitrogen probe sized molecule with a radius of 1.86 Å can access the pores for geometric surface area calculations.^1^ Fig. S1–S3 and Table S1 of the ESI† show the CSD refcodes and more detailed information on the excluded MOFs. Fig. 1 shows distributions of the geometric properties of MOFs and their evolution from 1995 to 2015; ESI† shows an animated version. While very few MOFs were known until the early 21st century, the dramatic increase in the number of structures from 2000 to 2015 is evidence of how the remarkable characteristics of MOFs enable the exploration of a wide range of physical properties in porous materials. Most MOFs are concentrated in regions with pore sizes < 10 Å and surface areas < 2000 m^2^ g^–1^, possibly due to the use of relatively inexpensive and commercially available short linkers such as terephthalic acid and the fact that this range of pore size is optimal for many gas storage and separation applications. As new synthesis methods of MOFs are designed every day, the introduction of longer linkers, more sophisticated SBUs and new topologies have continued increasing during the past decade.^40^

## Identification of target MOF families

We used ConQuest in the CSD MOF subset to identify MOFs with the desired SBUs; ConQuest offers the user a wide range of flexible search options based on the metal centers, organic linkers or SBUs. We developed search criteria for six prototypical MOF families well studied in the literature: Zr-oxide nodes (*e.g.* UiO-66), Cu–Cu paddlewheels (*e.g.* HKUST-1), ZIF-like, Zn-oxide nodes, IRMOF-like, and MOF-74/CPO-27-like materials. We also devised search criteria to identify MOFs containing common functional groups such as alkyls, alkoxys, halogens as well as polar functionalities, allowing to discriminate on the surface chemistry and therefore on the hydrophilic/phobic nature of the MOFs. We anticipate that these criteria introduce guidelines for MOF researchers to perform quickly targeted MOF searches, not only for the above classes of MOFs and surface chemistry but also for additional ones; criteria can be customized in ConQuest, as explained below, to look for new MOF chemistries.

**Fig. 2:**
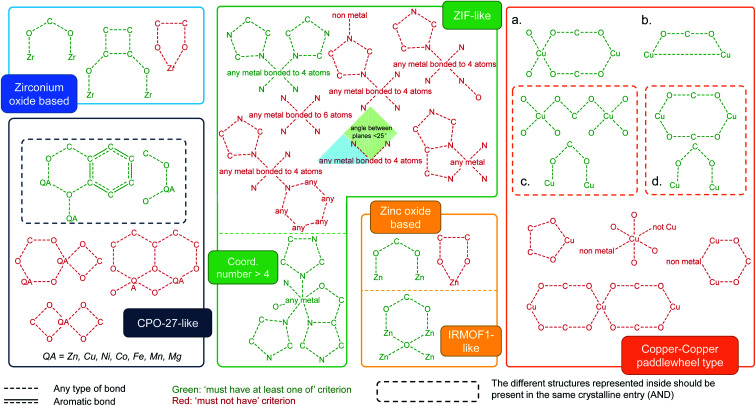
Criteria developed for the identification of MOF families in the CSD MOF subset based on specific secondary building units and their connection to the organic linkers. The target MOF families are zirconium oxide, MOF-74/CPO-27-like, ZIF-like, zinc oxide and IRMOF-like, as well as Cu–Cu paddle-wheeled materials. (a–d) Diagrams used to look for structures containing Cu–Cu paddlewheels. The dotted box for (c) and (d) means the structures inside should be considered as one single query. The red diagrams are queries used to eliminate undesired structures. See ESI† for more details on each MOF family.

Intuitively, our initial approach to look for specific MOF families was to fully draw and search for each SBU in ConQuest. Interestingly, this approach resulted in fewer than expected MOF hits in each category. This is because, when dealing with infinite polymeric structures, ConQuest carries out its searches on the smallest repeating unit based on the crystallographic symmetry, which may be different from the desired SBU, and therefore missing out MOFs where the full metal cluster is not represented. In other words, complete metal cluster information is only “assembled” in full when the unit cell is requested. To overcome this challenge regarding cluster representation, we developed a series of criteria to ensure that even partially represented MOF secondary building units are included in our search. Fig. 2 summarizes the criteria developed for the identification of each MOF family. We used a step-by-step approach, where we started from the simplest search for a MOF family and then gradually tuning the search criteria by including or excluding certain bonds and connections in the metal cluster. At each step, the resulting materials were constantly inspected until all unwanted structures were removed and target MOFs were identified. The green and red diagrams included in Fig. 2 represent search queries in ConQuest that are respectively labeled as “must-have” and “must not have” queries. A criterion for a target MOF family is either one single “must-have” query, such as IRMOF-like structures, or a combination of “must-have” and “must not have” queries. When several “must-have” queries are represented separately, they correspond to an OR statement, and therefore only one of the green diagrams is required to be present in each search hit (see for example the Zr-oxide-based family in Fig. 2). When several “must-have” queries are represented in the same dotted box, they correspond to an “AND” statement, and therefore each search hit should contain all the green diagrams (see MOF-74/CPO-27-type in Fig. 2).

We showcase here the derivation of the four search criteria for the family of Cu–Cu paddlewheel MOFs, which are a good example because they are usually not fully represented in ConQuest; Fig. S4–S17† show the derivation of criteria for other MOF families. Fig. 2a represents the diagram of one complete paddlewheel and its connection to the linker *via* the two oxygen atoms. However, there are multiple cases where only half of the paddlewheel is represented. These structures are found using Fig. 2b diagram, which contains only a section of the paddlewheel. We omitted the oxygen atoms from the linker, as we found that keeping these atoms returns fewer target structures. In this case, the two copper atoms are now bonded, corresponding to the rotational axis of the paddlewheel. More structures were found using the search criterion shown in Fig. 2c diagram, which is in turn comprised of two parts. The upper part brings in structures in which the represented paddlewheel is “broken”. However, other Cu-based structures with linear linkers are also included; this is avoided by adding the lower part, which represents the connection between the metal atoms and the linkers. The upper part of Fig. 2d diagram is similar to the diagram in Fig. 2a, without the oxygen atoms from the linkers bonded to the Cu atoms. Together with the lower part of the search criterion, the diagram from Fig. 2d captures structures where the paddlewheel and the metal-linker connections are represented separately in ConQuest. Fig. S10† shows the structure hits. All in all, the four “must-have” queries result in 1426 structures, some of which are not of the target type. To filter out these unwanted structures, we included another set of “must not have” criteria according to specific undesired structures (Fig. S11†). The combination of the “must-have” and the “must not have” criteria leads to a total of 1015 MOFs containing Cu–Cu paddlewheel building blocks.

In order to extend a targeted search, we encourage MOF researchers to access these groups of MOFs and use the “combine queries” function in ConQuest for browsing and search analysis of other desired structures in the CSD MOF subset. Looking at the selected families shown in this work, Fig. S28† shows the comparison of the geometric properties and the number of structures in each MOF category. Combined together, Zn-oxide and IRMOF-like materials account for 3187 structures, followed by 1015 for Cu–Cu paddlewheels, 274 for ZIFs, 108 for CPO-27-like structures and 77 for Zr-oxide structures in the CSD 5.37 version from May 2016.

## Identification of surface functionalities in MOFs

**Fig. 3:**
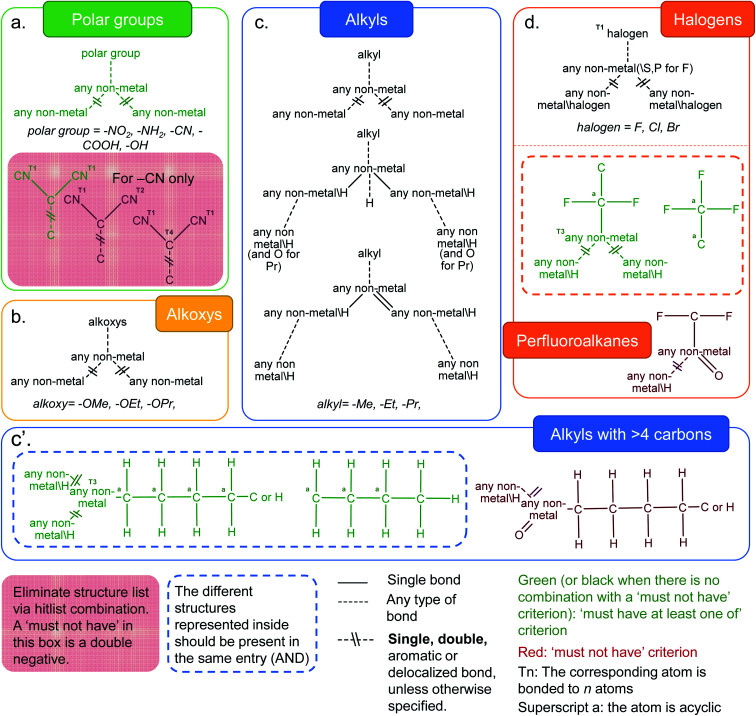
Criteria developed to identify MOFs with common functionalities in the CSD MOF subset. (a) Polar groups (–NH_2_, –NO_2_, –CN, –COOH and –OH). For the –CN case, the red box represents queries which target dicyanides that are chosen to be eliminated. This dicyanide search is obtained *via* a combination of one “must-have” query and two “must not have” queries. The green diagram is thus an overall negative and the red diagrams are double negatives; (b) alkoxys (methoxy, ethoxy, propyloxy); (c) alkyls (methyl, ethyl, propyl); (c′) alkyls (with more than 4 carbon atoms on the left) and (d) halogens (–F, –Cl, –Br), and structures with perfluoroalkane groups. The variable bonds are all the same type for queries within the grey dotted box: single, double, aromatic or delocalized. For the three queries outside of the grey dotted box, the variable bonds are either aromatic or delocalized. See ESI† for more details on each functional group.

Functionalization plays a crucial role in fine-tuning the chemical and physical properties in MOFs. Rational incorporation of chemical functionalities has been extensively employed using various pre- or post-synthetic engineering techniques as well as in computer models of MOFs for a breadth of applications including carbon capture,^41^,^42^ gas separation and sensing,^43^–^45^ catalysis,^46^,^47^ light harvesting^48^ and optical luminescence.^49^ We have considered a number of distinct functional groups categories such as polar functional groups (–NH_2_, –NO_2_, –CN, –COOH, –OH), alkoxys (methoxy, ethoxy, propyloxy), alkyls (methyl, ethyl, propyl and alkyls containing more than 4 carbon atoms) and halogens (–F, –Cl, –Br). Fig. 3 shows the combination of ConQuest queries used, together with the CSD Python API scripts, to target these functionalized MOFs. The use of the CSD Python API makes it possible to ensure that the search fragments are only present in the main framework and not part of a solvent. The Python script is available in the ESI; Fig. S28–S32† show the frequency of occurrence as well the geometric properties for all MOFs with the functional groups described above.

## Identification of chiral MOFs

**Fig. 4:**
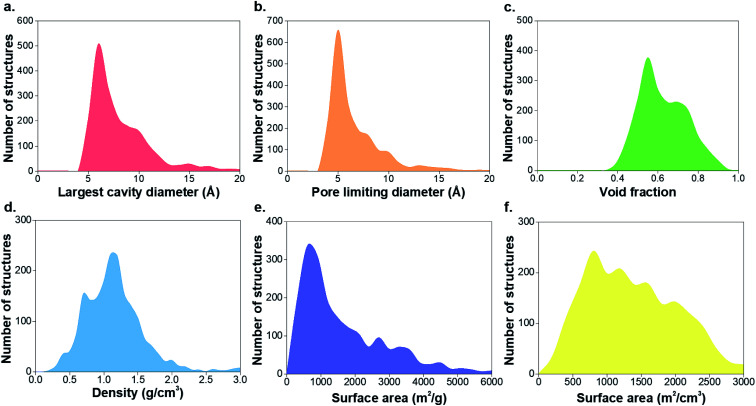
Histograms of the geometric properties of 1911 chiral structures with non-zero gravimetric surface area in the CSD MOF subset. (a) Largest cavity diameter, (b) pore limiting diameter, (c) void fraction, (d) density, (e) gravimetric surface area, (f) volumetric surface area.

Many of the target subsets of MOFs explored in this work are closely related to adsorption-based applications, which also guide the criteria to design the queries to identify these subsets. Thus, the list of 55 547 structures in the CSD MOF subset narrows down to 8253 porous MOFs. Similarly, considering other type of applications from a wider range of areas, we can tune these queries according to a new set of criteria and design a different subset suitable for these purposes. As an example of this, precise knowledge of existing chiral MOFs and their structural properties facilitates the identification and engineering of MOF chirality for niche catalytic and enantio-separation applications.^14^,^50^–^52^ Given the flexibility provided by CSD Python API scripts, we also included chirality of MOFs. Here, we defined a chiral MOF when it presents either chiral atoms in the structure or a chiral crystal packing. We found 4504 structures containing *S*/*R*-chiral atoms and 6859 structures in Sohncke-chiral space groups; combinatorial searches of chiral-ligand MOFs in chiral space groups gave 2010 structures. It should be noted that we focused on *R*/*S* chirality and therefore structures with *e.g.* metal lambda/delta or axially-chiral structures were not accounted for. Fig. 4 shows the physical and geometric properties for 1911 chiral structures with non-zero surface area values. This study brings some interesting historical insights. The group of chiral porous MOFs is included in the 8253 porous MOF subset and comprises around a 23% of the latter. As a result, the distribution of geometrical properties is similar, and the majority of chiral structures synthesized so far contain small pores of < 10 Å and surface area values of < 2000 m^2^ g^–1^. However, non-porous structures are only ∼5% of the whole group of chiral MOFs, which suggests the fact that researchers were actually looking for porous chiral structures. This is connected to the fact that more of 90% of chiral MOFs were synthesized after the 2000s, when MOFs started growing as a field, to explore their potential for catalytic applications and enantiomeric resolution.

## Porous network connectivity and framework dimensionality

**Fig. 5:**
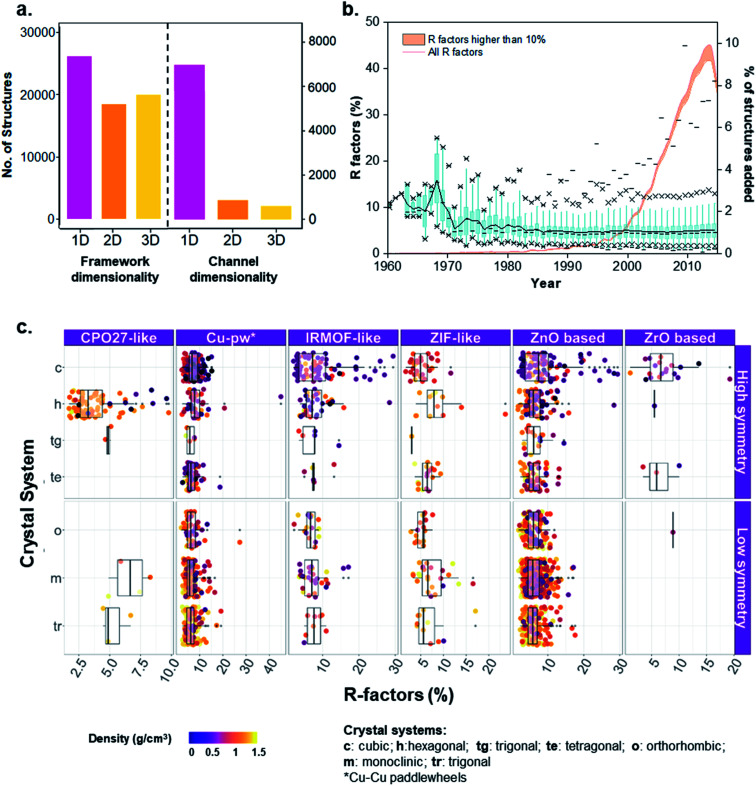
Analysis of MOFs included in the CSD. (a) Histograms of framework and channel/pore dimensionalities characterized for the 52 787 structures. (b) Non-cumulative evolution of R factors of the MOF subset from 1960 to 2015. Blue: boxplots of *R*-factors per year. Percentiles used: 1% (lower dash symbol), 25% (lower cross symbol), 50% (dash in the box), 75% (upper cross symbol), 99% (upper dash symbol). A black line connects the means across all the boxes; the orange curve shows the percentage of structures added to the database per year. The orange area under the orange curve highlights the number of structures with an *R*-factor higher than 10%. (c) Distribution of *R*-factors and density across different MOF families and crystal systems of low or high symmetry.

Knowing the porous network connectivity or dimensionality (also referred to as percolation) is important in determining MOFs applicability in certain adsorption applications. For example, 1D channeled MOFs have shown to be highly selective in the separation of hydrocarbons due to favorable thermodynamic or kinetic origins towards one component, depending on channel size and shape.^53^–^55^ The diverse nature of building units' linkage in MOFs results in variations of porous networks, where the connectivity of a porous network is determined by a geometric analysis of connecting pathways of porous components, resulting in 1D channels and 2D or 3D networks. Porous networks are normally sampled using mesh/grid-based propagation techniques that map the void space into connected components.^56^–^59^ To investigate the pore system accessibility and dimensionality, we used Poreblazer,^59^ a freely available set of tools for the structural characterization of materials, to determine the geometrical parameters of the pore networks for all 8253 porous structures in the MOF subset. Fig. 5a shows the analysis, resulting in 86% 1D, 9% 2D and 4% 3D pore connectivity for these porous structures. The corresponding refcodes are provided in the ESI.†

In addition to the pore network, framework dimensionality is also critical for selecting an optimal MOF for a given application. Whereas having a large landscape of structures helps to set up a global point of view on property–performance relationships, the dimensionality of the structure will help to decide which material is more practical. As previously explained, we used our in-house developed script for the determination of the framework dimensionality. The results for all 52 787 porous and non-porous MOFs are included in Fig. 5a, where 40% of the structures are 1D, 29% are 2D and 31% are 3D. The corresponding refcodes are provided in the ESI.†

## An insight into quality crystals of different MOF families

When dealing with such a high amount of experimental data, it is useful and interesting to have a better idea of the data quality. A simple way of assessing the quality of crystal structures is to analyze their crystallographic *R*-factors, available in the CSD database and extractable *via* the CSD Python API. High *R*-factors, typically above 10%, reflect refinement models that may contain systematic errors.^60^Fig. 5b shows the evolution of the *R*-factors of the MOF materials from 1960 to 2015; Fig. S34 and S35† show the characterization of the physical and geometric properties for all MOFs and the corresponding families *vs. R*-factors. Although the field of MOFs is generally considered to have started in the late 1990s (ref. 61 and 62) – as reflected by the increasing number of structures on Fig. 5b, scientists have been working on coordination polymers since the late 1950s, and even before. However, since the definition of MOFs is still debated today,^1^,^63^,^64^ it is not straightforward to tell which structure truly is the first MOF. The oldest structure in the CSD MOF subset dates back to 1940 and consists of a sodium formate (NAFORM).^65^ The general opinion would hardly consider this a MOF nowadays, although it still marginally fits the criteria required for being part of the CSD MOF subset. The most ‘MOF-like’ 3D coordination polymer structure from the early days must be ADINCU by Saito and coworkers from 1959,^66^ which is widely recognized by the community. This work was followed by Hoskins and Robson (JARMEU) and then by the groups of Yaghi and Kitagawa. We have, therefore, started our timeline in 1960. Despite the fact that the number of structures with *R*-factors higher than 10% has increased over the last decade, reaching 0.7% of the MOF subset in 2013, the mean and the median *R*-factor values have remained fixed at around 5%, and 99% of the structures have *R*-factors lower than 12%. To understand the evolution, it is worth noting the technological advances in crystal structure determination between the 1960s and today. Until the 1970s, the mean values for most structures are above 10%, while in the 1980s, the *R*-factors significantly dropped to below 10% despite the increase in more complex and large structures being synthesized.^67^

The development of MOF families such as the ones introduced above enables data analyses that provide an overview of the properties dedicated to these smaller subsets. As an example, Fig. 5c explores the quality of MOF structures – *via* their *R*-factors – by looking at their family (*e.g.* IRMOF-like, ZIF, *etc.*), crystal system, symmetry and density. For each family, structures are divided into their crystal systems and a boxplot shows the distribution of their *R*-factors. The crystal systems are arranged in decreasing order of symmetry: cubic, hexagonal, trigonal and tetragonal systems considered as “high symmetry”, and orthorhombic, monoclinic and triclinic considered as “low symmetry”. Each point representing a structure is then colored according to its density. The property-landscape provided here shows for example that some families crystallize in specific crystal systems (see CPO-27/MOF-74 and Zr-oxide MOFs), whereas others crystallize in all crystal systems, with different distributions. For instance, IRMOF-like structures tend to crystallize mainly in cubic or hexagonal systems and show higher *R*-factors in these systems. In general, the data presented here suggests that for all the families, low-density MOFs tend to form high symmetry structures – in accordance with the analysis of Øien-Ødegaard and co-workers.^60^ From the general overview given in Fig. 5c, it is possible to focus on more specific aspects of *R*-factors for each family. For example, the boxplots in Fig. S36† show the distribution of *R*-factors among each crystal system for each family; those in Fig. S38† show the distribution of *R*-factors among high and low symmetry structures for each family.

An artificial way of “correcting” the experimental values obtained from X-ray diffraction patterns is to mask the solvent. To explore the effect of solvent masking on the quality of the crystal structure data, we finally compared the role of the structure refinement software SQUEEZE^68^ in the distribution of *R*-factors. SQUEEZE enables users to identify and include the contribution of disordered solvent in the calculated structure factors upon determination of the crystal structure. Fig. S39† shows boxplot representation of the *R*-factors for the different MOF families, comparing the values on structures that have had their solvent masked through SQUEEZE and those that have not gone through this process. Although it might seem simple to assume that the use of SQUEEZE will lead to lower *R*-factors, there is not a clear trend to support this statement. One of the major difficulties when considering solvent masking and *R*-factors is how to determine what will produce the best structure for your purposes; a slightly lower *R*-factor structure that has had SQUEEZE applied, or a higher *R*-factor structure with an attempt to model all the disorder positions of the framework and/or guests.

It should be remembered that, although the *R*-factor is a convenient single metric to assess the quality of crystal structures, it simply measures the agreement between the refined model and the experimental data. The *R*-factor does not take into account how chemically and physically meaningful the resulting structure is, whether any use of solvent masking is appropriate or whether there are large residual electron density peaks. A more thorough analysis of the data quality in the MOF subsets will be addressed as part of future work.

## High-throughput simulation of hydrogen uptake at room temperature and high pressure

To demonstrate the usefulness of the methods and analysis presented in this paper, we included their application into hydrogen storage, using an HTS based on grand canonical Monte Carlo (GCMC) simulations. Cost-effective and high capacity hydrogen storage remains a challenge for the widespread use of fuel cell applications. Although hydrogen has a higher gravimetric energy density than most other fuels, its volumetric energy density is one of the lowest.^69^ The main challenge is thus to store enough hydrogen in a compact space. The US Department of Energy has set a target of 30 g L^–1^ of volumetric capacity by 2020 in order to ultimately reach 50 g L^–1^.^70^ Among the possible storage solutions being currently researched, adsorption in porous materials is a promising one. As current on-board containers operate at high pressures (700 bar for Toyota fuel cell vehicles) and room temperature,^71^ we predicted the adsorption uptake at 298 K over a range of low to high pressures of 200, 500 and 900 bars. Although high-throughput screening has been widely performed on MOFs for hydrogen storage, very little work published results at these conditions.^72^ In addition, the classification presented in this paper enables interesting visualizations regarding the performance of different classes of MOFs, thereby either further confirming previous observations with the amount of data available in the CSD MOF subset or presenting new ones. Using the methods described above, readers can also create their own classification and map it to their screening results.

**Fig. 6:**
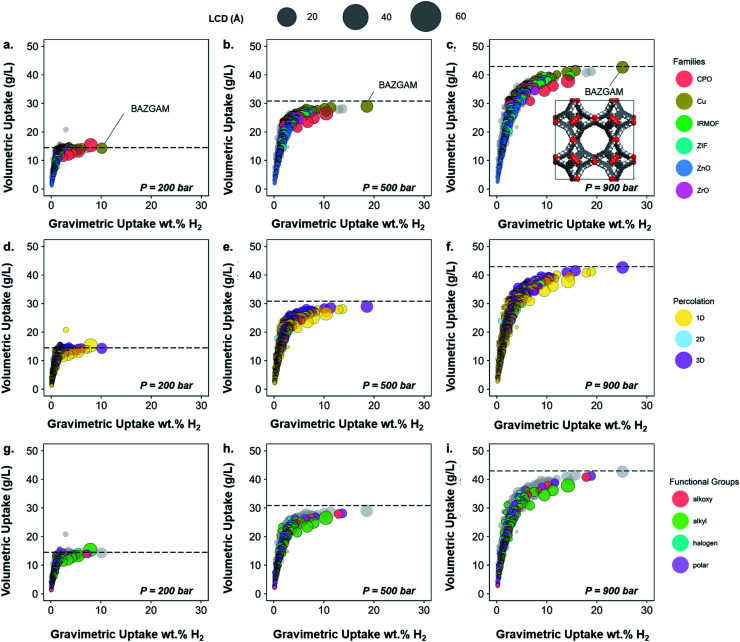
Characterization of the 3D MOFs screened for hydrogen storage. Volumetric uptake *vs.* absolute uptake wt% H_2_ at room temperature at 200, 500 and 900 bar. Each circle represents a MOF structure. The sizes of the circles represent the LCD in all plots. The dashed line corresponds to the volumetric uptake obtained in an empty tank. (a–c) Families of the screened structures; structures that have not been assigned a family are colored in grey in the background. The highlighted structure BAZGAM is shown in the inset at 900 bar. (d–f) Percolation of the screened structures. Structures containing 1D, 2D and 3D pore channels are respectively represented in yellow, blue and purple. (g–i) Functional groups identified in the screened structures. Structures that have no particular functional groups identified are colored in grey in the background. Full hydrogen adsorption data can be found online at ; http://aam.ceb.cam.ac.uk/mof-explorer/H2_HTS.

From the previously obtained 52 787 structures, we selected 13 738 structures with pores large enough for a hydrogen molecule to navigate through. To further prepare the structures for the HTS with hydrogen, we eliminated any remnant structures with non-missing hydrogen atoms but hydrogen-related disorder (see Methods section), which led to 6355 structures on which we performed the screening. Fig. 6a–c shows the volumetric uptake (mass of hydrogen over volume of framework) *versus* the gravimetric uptake (mass of hydrogen over total system mass) of these structures at the three considered pressures. Each circle represents a MOF. The colors highlight the six different families of MOFs chosen in this paper, as described above, whereas grey circles represent the structures that do not fit in this classification; Fig. 6d–f and g–i highlight the pore dimensionality and surface chemistry, respectively, of the structures. The size of each circle represents the largest cavity diameter (LCD) of the corresponding structure. The corresponding gravimetric uptake in an empty tank is represented with a dashed line. A dynamic representation of the simulations can be found at ; http://aam.ceb.cam.ac.uk/mof-explorer/H2_HTS. Similar to our previous work,^45^,^73^,^74^ this allows the visualization of hydrogen gravimetric and volumetric uptakes with respect to different structural properties such as void fraction, LCD, pore-limiting diameter (PLD), isosteric heat of adsorption, and surface area to better understand their role. More importantly, it allows the multidimensional visualization of the generated data in an interactive way, where, each data point (*i.e.* each MOF) can be individually identified and tracked into the CSD and the CCDC website.

The empty tank reference shows that, for pressures higher than 200 bar and at room temperature, the MOFs do not provide any improvement in terms of volumetric uptake. This analysis shows that room temperature and high pressure are not the way forward for efficient hydrogen storage in porous materials unless new radical ideas are implemented. Nevertheless, the trends obtained still unveil valuable insights; we will henceforth focus on the information gained from mapping our classification to the screening results.

Fig. 6a–c shows that the highest uptakes, especially gravimetric, are obtained for Cu–Cu paddlewheel, CPO-27/MOF-74-like and IRMOFs structures, whereas other Zn-oxide-type structures tend to have lower performance. Zr-MOFs, known to have large chemical stability among MOFs, show moderate gravimetric uptakes but competitive volumetric values. When looking at the pore connectivity, the trends reproduce those from the MOF families found here (Fig. 6d–f). In particular, Cu–Cu paddlewheel MOFs form 3D-pore networks whereas CPO-27/MOF-74 form 1D channels and therefore the highest uptakes are for 3D and 1D MOFs. Fig. 6g–i show that alkyl, alkoxy and polar groups are often present in high uptakes, whereas structures containing alkyl groups have a slightly lower volumetric uptake. Fig. S40† shows in more detail the nature of the functional groups in these cases: –CH_3_, –OH and –OCH_3_ are the functional groups present in the best-performing structures. Fig. S41 and S42† provide similar information with regard to the structures' crystal systems and the metal atoms they contain. Fig. S42† is particularly interesting when combined with Fig. 6a–c, as they suggest the best-performing CPO-27/MOF-74-type structures – which are among the overall best-performing ones – are frameworks containing magnesium atoms due to its lighter character. This is in agreement with studies on the role of magnesium in better hydrogen adsorption in MOFs.^69^ All in all, the structure with the best volumetric and absolute uptake is a Cu–Cu-paddlewheel, 3D-pore networked unfunctionalized MOF, BAZGAM (Fig. 6a–c), which has been identified previously in the literature for its exceptional performance at 77 K and 100 bar (reported values of 34.3 g L^–1^ and 19.3 wt% H_2_).^72^ At room temperature and 900 bar, its uptake values are 42.7 g L^–1^ and 25.1 wt% H_2_.

**Fig. 7:**
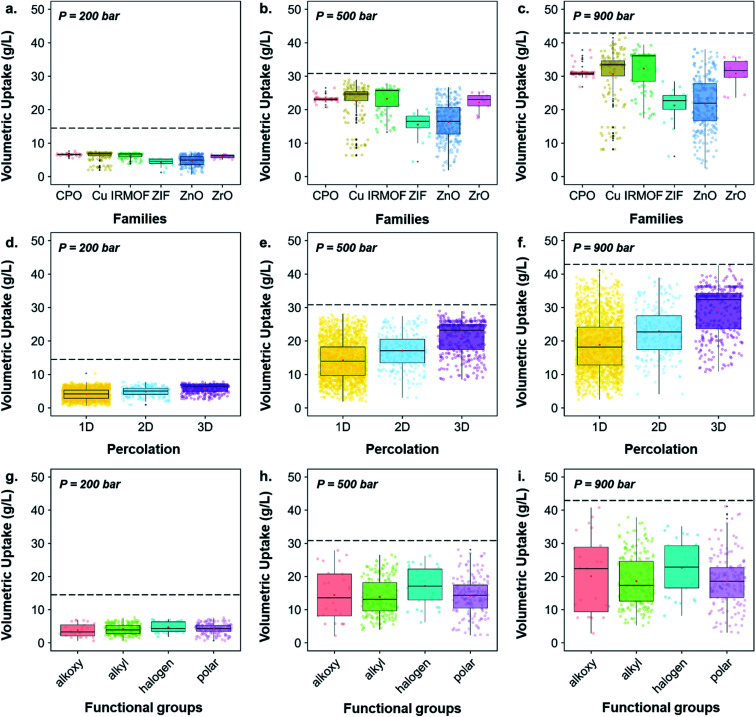
Quantitative characterization of the 3D MOFs screened for hydrogen storage boxplots of volumetric uptake of H_2_ at room temperature at 200, 500 and 900 bars *versus* (a–c) families of the screened structures, (d–f) percolation of the screened structures and (g–i) functional groups identified in the screened structures. The jittered points in the background give an idea on the number of structures considered for each boxplot. The markers represent the minimum, first quartile, median, third quartile, and maximum values, respectively. Outliers are represented by black data points. The dashed line corresponds to the volumetric uptake obtained in an empty tank.

While Fig. 6 highlighted the characteristics of the best-performing structures, Fig. 7 gives more quantitative insights, through statistical analyses, of these observations; Fig. S43† provides similar boxplots in terms of gravimetric uptake. Fig. 7a–c, d–f and g–i show boxplots representations of the volumetric uptake for each of the MOF families, the percolation and the type of surface chemistry present, respectively. Fig. 7a–c show that CPO-27/MOF-74-like, Cu–Cu-paddlewheels, IRMOFs and Zr-oxide MOFs perform better at all three different pressures. In addition, they adsorb hydrogen more easily as the pressure increases: the amount of hydrogen adsorbed in ZIFs and Zn-oxide-type structures quadruples from *ca.* 5 to 20 g L^–1^ as the storage pressure increases from 200 to 900 bar, whereas the amount adsorbed in CPO-27/MOF-74-like, Cu–Cu-paddlewheels, Zr-oxide and IRMOFs structures increases from *ca.* 7 to 30 g L^–1^, reaching 32 g L^–1^ in IRMOFs, over the same range of pressures. Interestingly, Fig. 7d–f show that 3D pore-network structures have, on average, higher volumetric uptake than 2D-channeled structures, which in turn have higher volumetric uptake than 1D-channeled structures. In addition, the difference in performance increases as the storage pressure increases: 3D-channeled structures have in average a 40, 48 and 53% higher uptake at 200, 500 and 900 bar, respectively, than 1D-channeled structures. Fig. 7g–i shows that structures containing halogen groups perform better overall, and the spread of volumetric uptake of structures containing alkyl groups is wider as the pressure increases. Fig. S40† provides a breakdown of each functional group, showing that structures containing –Br, –F and –OCH_2_CH_3_ groups stand out as having the highest volumetric uptakes.

Previous similar work that screened MOFs for hydrogen storage focused on the relationship between their geometrical properties (such as pore volume^75^ or void fraction^69^) and performance. In our case, we have mapped out the behavior of the different classes of MOFs outlined in this paper, thus providing a clearer picture of the CSD MOF subset landscape. In particular, we have identified the volumetric and gravimetric storage limits for different families of MOFs, thus offering more insights into which MOF space is more promising or lacking.

**Fig. 8:**
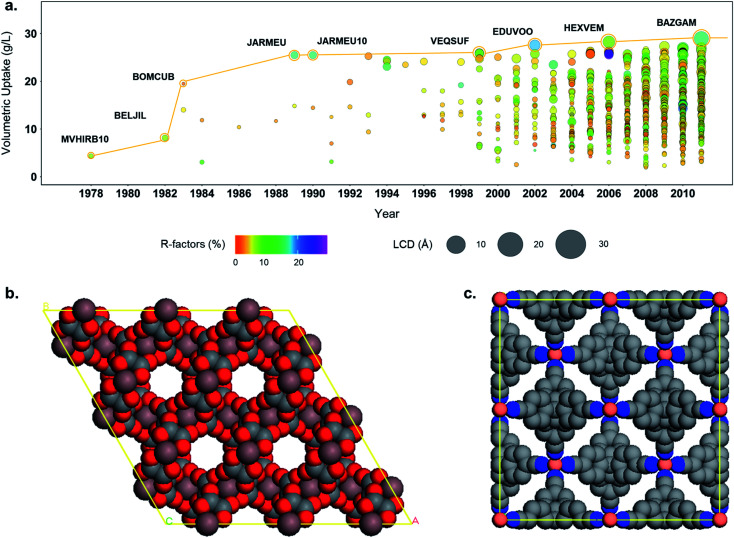
(a) Evolution of the structure with the highest hydrogen volumetric uptake at room temperature and at 500 bars in the CSD over the years. Each circle represents a structure. The size indicates the LCD, the color the corresponding *R*-factor. Each new best performing structure is highlighted with a yellow circle and the yellow line tracks the best performing structure over the years. (b) Snapshot of a supercell of BOMCUB. The counter-ions and water molecules were removed from the snapshot for clarity. (c) Snapshot of a supercell of JARMEU.

In addition to the structure–property relationships that can be uncovered from combining simulation data and the structural data available *via* the CSD and the developed subsets, the tools developed here allows a better understanding of the evolution of the MOF field. Fig. 8a shows the evolution of the hydrogen volumetric uptakes at room temperature and 500 bar for the 3D MOFs included in the CSD over the years. Each circle represents a MOF; their size corresponds to their LCD and the colors indicate their *R* factors. The yellow line traces the best-performing structure throughout time. Interestingly, the biggest jumps in terms of volumetric uptake – reaching 19.4 and 25.2 g L^–1^ – happened in 1983 and 1989, with structures BOMCUB^76^ and JARMEU,^77^ respectively, when only a few fairly good quality structures were submitted. Fig. 8b and c show the snapshots of these two structures: BOMCUB being an oxalate complex synthesized by Siftar and coworkers; and JARMEU being an infinite polymeric framework consisting of three dimensionally-linked rod-like segments synthesized by Hoskins and Robson. The number of structures then significantly increased in the late 1990s, with slightly higher *R* factors and higher LCDs. Starting from the 2000s, the *R* factors and LCDs become more varied and the highest volumetric uptake reaches a maximum of 28.8 g L^–1^.

## Outlook

The coordination geometry of inorganic units and the diverse nature of MOF linkers have given rise to the emergence of thousands of diverse MOF materials with currently over 99 000 structures present in the CSD MOF subset. Here, we developed a customized set of criteria to identify specific families of MOFs as a powerful tool to classify them and speed up the way MOFs are being investigated for different applications. The computational tools and the interactive online data explorers provided in this work will allow MOF researchers to browse and look for targeted MOF categories based on secondary building units, chirality, surface chemistry as well as geometrical properties including pore and framework dimensionality. Through CCDC's structure search program ConQuest, the principles we supplied here allow users to search for and identify new MOF families and functionalities based on any of the diverse pool of MOF building blocks. We also show the usefulness of these tools with a high-throughput screening for hydrogen storage at room temperature using grand canonical Monte Carlo simulations. On the one hand, the interactive website allowed the visualization of the multidimensional influence of different parameters and the identification of each data point in the CSD, together with the original publication of the structure. On the other hand, the statistical analysis quantifies the impact of the structural descriptors on the performance. We expect that this work will guide experimentalists and theoretical researchers to probe the chemistry of MOFs for transformative advances in their applications.

## Methods

### MOF explorer for 5D exploration of structural properties

All family-property relationships of the 8253 porous MOFs presented in this work can be found online at http://aam.ceb.cam.ac.uk/mof-explorer/CSD_MOF_subset. Hydrogen adsorption data can be found online at ; http://aam.ceb.cam.ac.uk/mof-explorer/H2_HTS. Users can explore the structural features and adsorption performance of porous MOFs interactively with any one of up to 18 variables plotted in 5 dimensions. Since data has been gathered for multiple MOF families and types, this leads to thousands of unique plots that can be generated according to the user's interest. MOF can be searched for and filtered by name, or by selecting them from the graph, allowing the user to track particular MOFs' characteristics.

### Structures preparation for high-throughput hydrogen uptake simulations

3D structures were selected from the CSD version 5.37 using the Python API script described above. All structures had their unbound solvent removed using the CSD Python API scripts published previously. Structures containing Cu–Cu paddlewheels and CPO-27/MOF-74-like structures had their bound solvent removed using the same scripts. Missing hydrogens were added using the add_hydrogen function in the CSD Python API. Any additional hydrogen-related disorder was removed by using the ‘non-disordered’ filter in ConQuest, following the protocol described recently to differentiate between the ‘non-disordered’ filter and the non-disordered MOF subset.^78^ A PLD of 2.8 Å, corresponding to the lowest *σ* of the hydrogen atom across different force fields, was used to eliminate structures with lower PLDs.

### Grand canonical Monte Carlo simulations

The GCMC simulations were performed in the multi-purpose code RASPA.^79^ We used an atomistic model of each structure where the framework atoms were kept fixed at their crystallographic positions. We used the standard Lennard-Jones (LJ) 12-6 potential to model the interactions between the framework and fluid atoms. In addition, a Coulomb potential was used for fluid–fluid interactions. The parameters for the framework atoms were obtained from Dreiding Force Field (DFF)^80^ and, when not available, from the Universal Force Field (UFF),^81^ whereas the hydrogen molecule was modeled by placing a single LJ sphere at the center of mass (see provided RASPA files in the ESI†).^82^ The Lorentz–Berthelot mixing rules were employed to calculate fluid-solid LJ parameters, and LJ interactions beyond the cutoff value of 12.8 Å were neglected. The simulation box for each structure is defined so that the cell lengths are larger than twice the cutoff distance. 30 000 Monte Carlo cycles were performed, the first third of which were used for equilibration and the remaining steps were used to calculate the ensemble averages. Monte Carlo moves consisted of insertions, deletions and displacements. In a cycle, *N* Monte Carlo moves are attempted, where *N* is defined as the maximum of 20 or the number of adsorbates in the simulation box. To calculate the gas-phase fugacity we used the Peng–Robinson equation of state.^83^

## Conflicts of interest

There are no conflicts to declare.

## Supplementary Material

Supplementary information
