# Supplementary material for: Targeted classification of metal–organic frameworks in the Cambridge structural database (CSD)
Source: Chem Sci. 2020 Jun 17;11(32):8373–87. doi: 10.1039/d0sc01297a (PMC7690317; doi:10.1039/d0sc01297a)
Supplement: Supplementary file 1 [file SC-011-D0SC01297A-s001.pdf]

Electronic Supplementary Material (ESI) for Chemical Science.
This journal is © The Royal Society of Chemistry 2020

Supplementary Information for:

Targeted Classification of Metal-Organic Frameworks in the Cambridge Structural Database (CSD)

Peyman Z. Moghadam,[a,‡,†,*] Aurelia Li,[a,†] Xiao-Wei Liu,[a,b,d] Rocio Bueno-Perez,[a] Shu-Dong Wang,[b] Seth B. Wiggin,[c] Peter A. Wood,[c] and David Fairen-Jimenez[a,*]

[a]Adsorption & Advanced Materials Laboratory (AAML), Department of Chemical Engineering & Biotechnology, University of Cambridge, Philippa Fawcett Drive, Cambridge CB3 0AS, UK

[b]Dalian National Laboratory for Clean Energy, Dalian Institute of Chemical Physics, Chinese Academy of Sciences, 457 Zhongshan Road, Dalian 116023, P. R. China

[c]The Cambridge Crystallographic Data Centre, 12 Union Road, Cambridge, UK

[d]University of Chinese Academy of Sciences, 19A Yuquan Road, Beijing 100049, P. R. China

*Correspondence and requests for materials should be addressed to D. F.-J. (df334@cam.ac.uk)

‡Current address: Department of Chemical and Biological Engineering, University of Sheffield, Mappin Street, Sheffield S1 3JD, UK. E-mail: p.moghadam@sheffield.ac.uk.

† These authors contributed equally.

Table of contents

S1. CSD MOF subset preparation prior to structural analysis 2
S2. MOF families classification: description of the criteria developed 5
S3. MOFs containing functional groups 16
S4. Calculations of MOFs physical and geometrical properties, dimensionalities of frameworks and channels 22
S5. Quality assessment of the data in the MOF subset using R factors 26
S6. GCMC simulations 29
S7. Database adjustment and general guidelines and updates 35
S8. References 36

S1. CSD MOF subset preparation prior to structural analysis

Based on the MOF subset in the CSD version 5.37,[1] a total of 55,547 non-disordered MOF materials were analyzed for their physical and chemical properties. The unbound solvents were removed for all structures – using previously developed Python scripts –[1] prior to the calculations. The bound solvents were removed for a total of 739 materials containing Cu-Cu paddle-wheels as well as CPO-27/MOF-74-like structures. We performed a number of checks for these 55,547 materials. Using automated algorithms, we identified and excluded 583 additional MOFs with structural disorder and 2,177 structures with missing framework hydrogens (see Figures S1-S3). The list of these "faulty" structures are given in the spreadsheet as supporting information. After removing these structures, 52,787 remaining MOF materials were evaluated for their geometrical and structural properties.

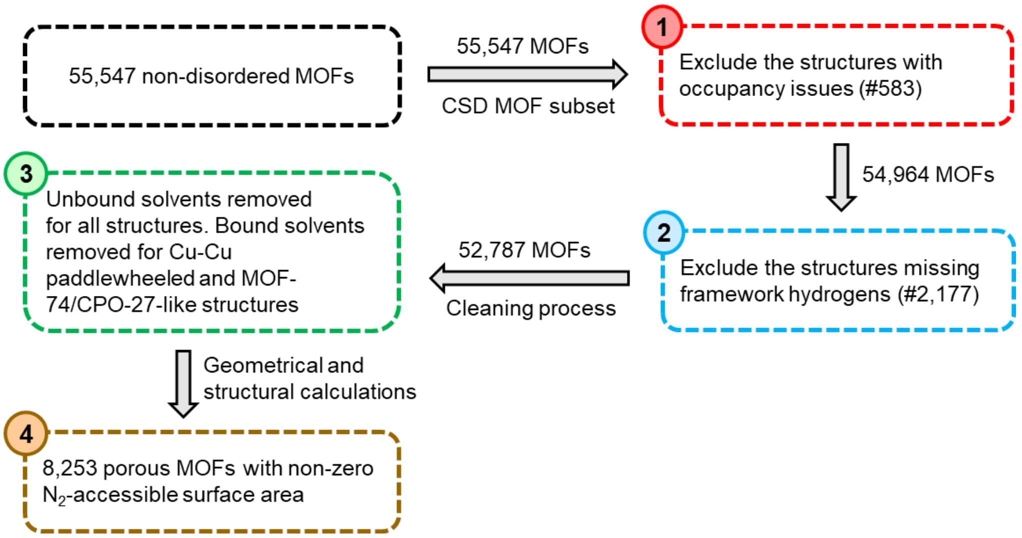

Figure S1. Flowchart outlining the CSD MOF subsets structures preparation prior to geometrical and structural calculations.

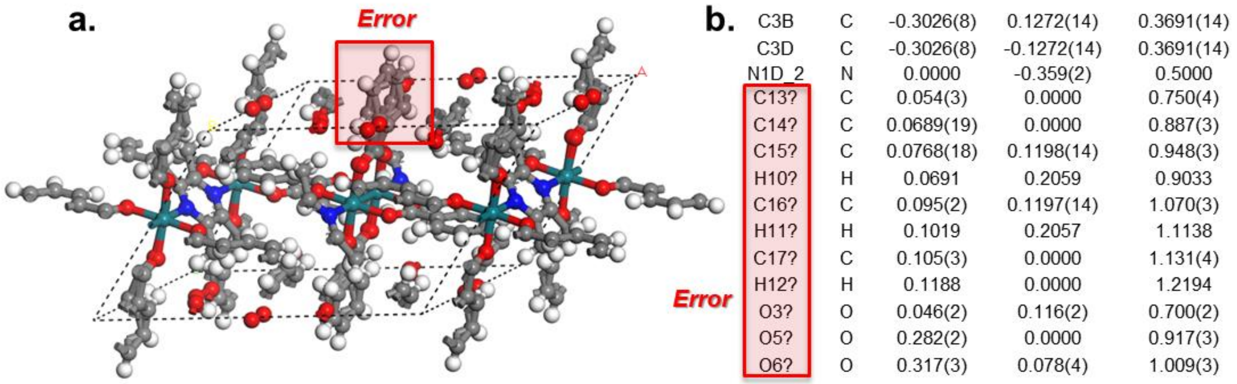

Figure S2. **a.** An example structure in the CSD MOF subset with occupancy issues (refcode: CIYER) **b.** atoms with occupancy issues in CIYER cif file are highlighted.

The shell script below was developed to automatically look for structures with occupancy errors or missing framework hydrogens:

```
for file in /urpath/urfiles*            # check all the cif files in your directory
do
    a=""                                # initial value
    grep -qP '\*|\?' "$file" && { a="+C"; sed -ri  '/\*|\?/d'  "$file"; }     # check "?" marks in the cif files; create suffixes to be added to the names of the structures
   a+=$(sed -n
 '/atom_site_fract_z/,${/H/p};/_chemical_formula_moiety/{N;s/\n//;/H/p}'     "$file"     |awk
'BEGIN{s[1,1]=0;s[0,0]=1;s[1,0]=2;s[0,1]=3}/_chemical_formula_moiety/{a=1;next}{b=1}END
{print "+"s[a++,b++]}')     # check the two zones in the cif files; create different suffixes to be added to the names of the structures
    echo ${file}${a} >>/urpath/report.txt     # output the results to report.txt
done
```

The script probes two zones in cif files to look for MOFs with missing hydrogens (Figure S3); in Zone 1, the chemical formula of the structure is specified (see the red box), and Zone 2 contains the information from the first line after '_atom_site_fract_z' to the end of the cif file (see the blue box). When a structure contains the H atom in Zone 1 but not in Zone 2, the structure was identified as a missing hydrogen framework. Note that in the case of MOFs with no H atoms in their chemistry (e.g. FMOF-1), "H" is not present in either zones.

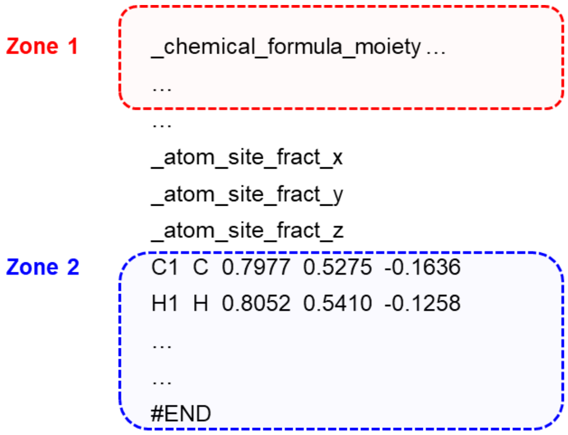

Figure S3. Zones inspected in all MOF cif files to look for missing H atoms in the framework.

Table S1. Different cases of suffixes to identify MOFs with occupancy errors or missing hydrogen atoms

Suffixes	Cases	Notes
+C	Contain “?” marks in the cif file	Wrong, removed
+0	Both Zone 1 & Zone 2 contain “H”	Correct structures
+1	Both Zone 1 & Zone 2 don’t contain “H”	Correct structures, e.g. FMOF-1
+2	Zone 1 contains “H”, Zone 2 doesn’t	Wrong, removed
+3	Zone 1 doesn’t contain “H”, Zone 2 does	Correct structures, “H” checked in Zone 2 are just the element labels, e.g. C_A, C_B … C_H, to distinguish the different situations of an element atom

S2. MOF families classification: description of the criteria developed

All searches were carried out in the MOF subset developed in our previous work based on CSD version 5.37 with May 2016 update.[1] The number of hits obtained for each criterion is given for all the MOF structures, ordered and disordered alike, in this document. The number of MOF hits along with their corresponding CSD refcodes for materials containing no structural disorder and also in porous MOFs are given in the Excel sheet as supporting information. Unless specified, all dotted bonds represented in the following queries are of "any" type. Green diagrams represent "must have" criteria, and red diagrams represent "must not have" criteria as explained in the paper.

Zr-oxide based MOFs

The Zr atoms in Zr-oxide-based MOFs such as UiO-66 (Figure S4) are bonded to the oxygen atoms of the carboxylic linkers. The circled area in Figure S4 shows the connection between the metal cluster and the organic linker. This specificity is expressed in our first "must have" criterion in Figure S5a. Figure S5b refers to the special case of Zr-oxide structures containing squarates in their linkers, in which case the two oxygen atoms of the carboxylic linker are bonded to two carbon atoms that are part of a four-atom ring. Only one such structure was found in the MOF subset. The combination of these searches returns 85 hits, of which some are not the target structures (see Figure S6). The criteria shown in the red diagram eliminates all these undesired hits, leading to a total of 77 Zr-oxide-based MOF structures.

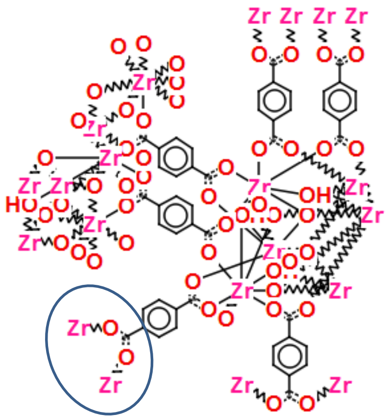

Figure S4. An example Zr-oxide based MOF; UiO-66, CSD refcode: RUBTAK02. The circle highlights the part of the MOF described by the criterion in Figure S5a.

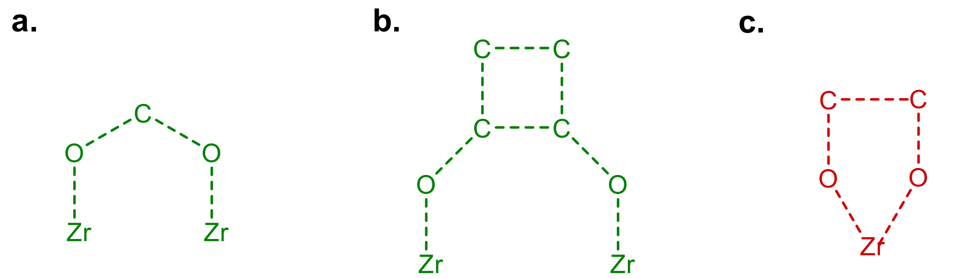

Figure S5. Criteria used to look for Zr-oxide based MOFs. **a.** and **b.** "must have" criteria, **c.** "must not have" criterion.

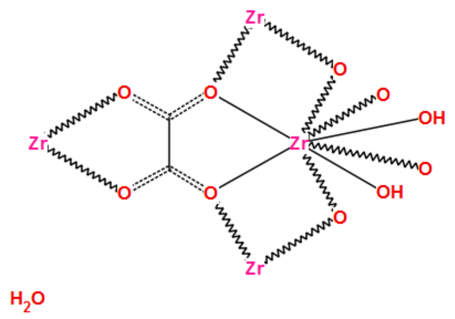

Figure S6. An example structure eliminated by the use of “must not have” criterion shown in Figure S5c. CSD refcode: VIXGAM.

Zn-oxide based MOFs

The Zn-oxide based MOFs is a family of which IRMOF-1 (MOF-5) is a member (Figure S7a). The diagrams presented in Figure S8 were developed using the same approach as for the Zr-oxide based MOFs. The combination of criteria results in 3,187 structures including IRMOF-1 materials. Figure S7b shows an example of a MOF with Zn-oxide SBUs (CSD refcode: ACOCUS).

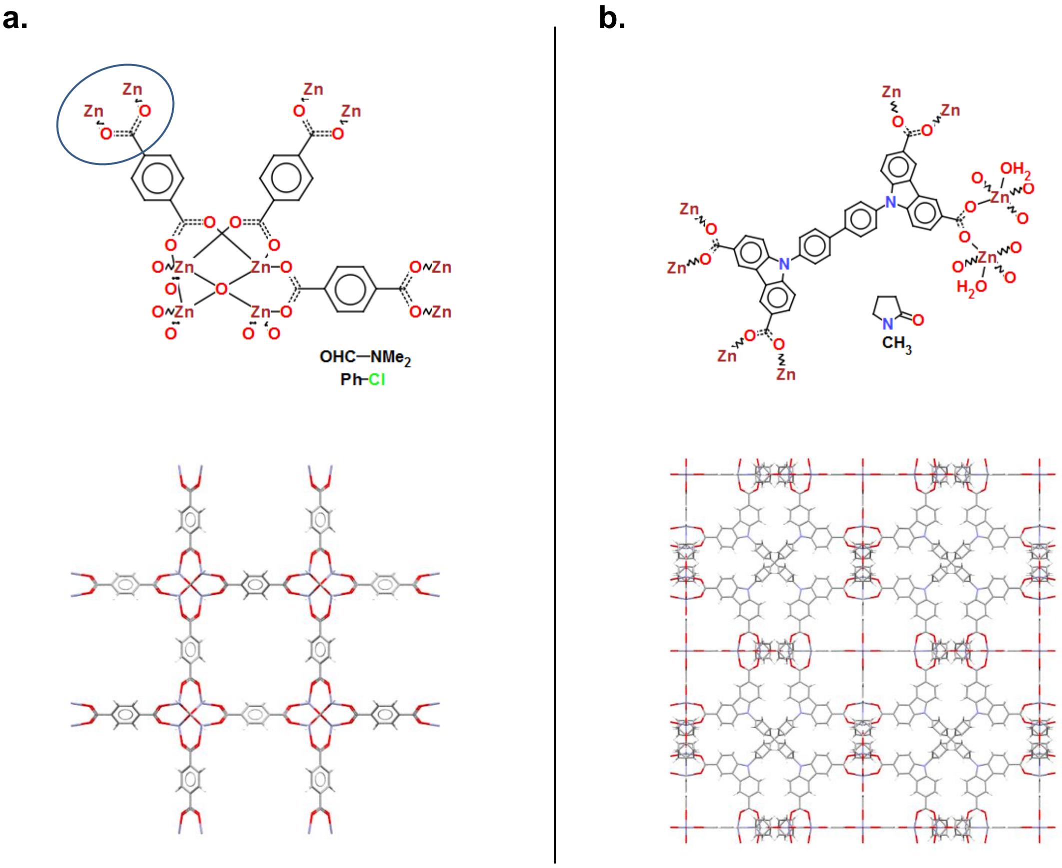

Figure S7. a. The structure of IRMOF-1 (MOF-5); CSD refcode: SAHYIK. The circle highlights the area captured by the criterion shown in Figure S8a. **b.** the structure of an example MOF with Zn-oxide SBUs. CSD refcode: ACOCUS.

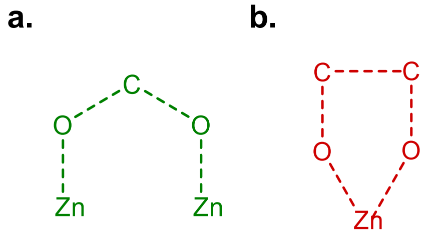

Figure S8. Criteria used to look for Zn-oxide based MOFs. **a.** “must have” criterion, **b.** “must not have” criterion.

To specifically look for IRMOF-like structures, another criterion was developed (Figure S9). Since the main difference between these MOFs and the more general Zn-oxide MOFs is the shape of the cluster, the criterion in Figure S9 was obtained from further customization of the metal node description in Figure S8a. 354 structures are returned for this criterion.

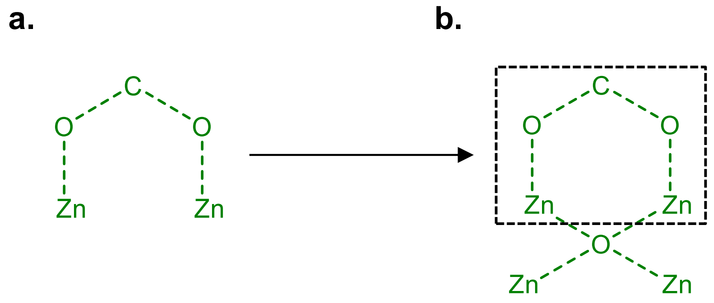

Figure S9. Derivation of criteria for IRMOF-like structures from the previous Zn-oxide-based structures criteria. Starting from **a.**, which is the “must have” criterion for the Zn-oxide based MOFs shown in Figure S8a, the metal cluster part is further described by including two additional Zn atoms and an oxygen atom, leading to criterion shown in **b.** The dotted box shows the same area in both criteria.

Cu-Cu paddle-wheeled MOFs

Upon examination of the hitlists obtained with criteria from Figure S10, some undesired structures needed to be eliminated. They were drawn according to the specific unwanted structures shown in Figure S11. The combination of the “must have” criteria represented in green in Figure S10 and the “must not have” criteria in red in Figure S11 leads to 1,015 structures with Cu-Cu paddle-wheeled SBUs (e.g. HKUST-1).

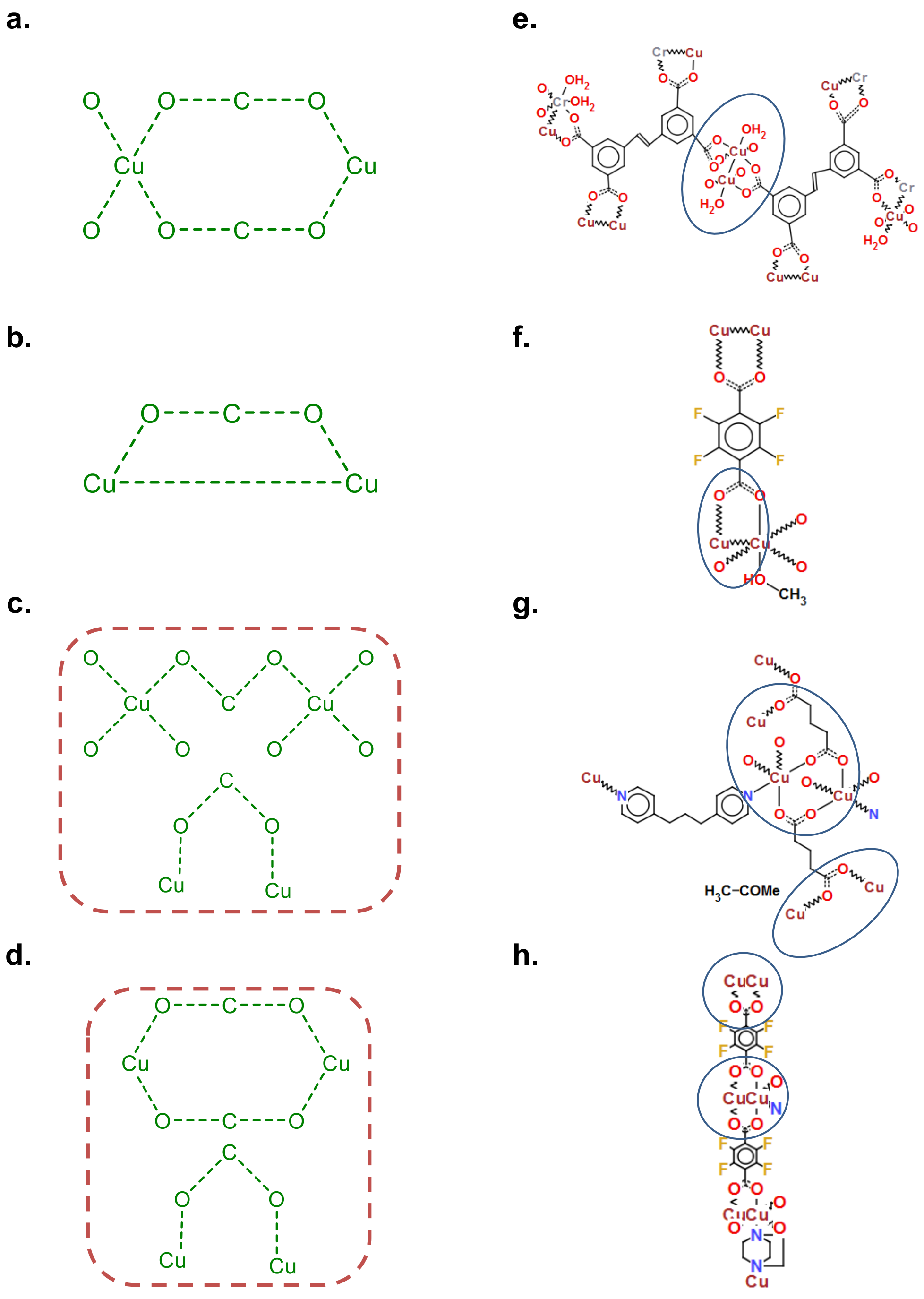

Figure S10. a. to d. Criteria developed to look for structures containing Cu-Cu paddlewheels. **a.** returns 988 hits, **b.** returns 611 hits, adds 178 to the list, **c.** returns 716 hits, adds 248 to the list, **d.** returns 647 hits, adds 12 to the list. For **c.** and **d.**, the dotted box means the structures inside should be considered as one single query. **e. to h.** Example structures found using the criterion on the left. The blue circled areas show the parts that have been searched for in ConQuest. CSD refcodes: **e.** ACUJOZ, **f.** ACASUT, **g.** ACAJOF, **h.** ACATAA.

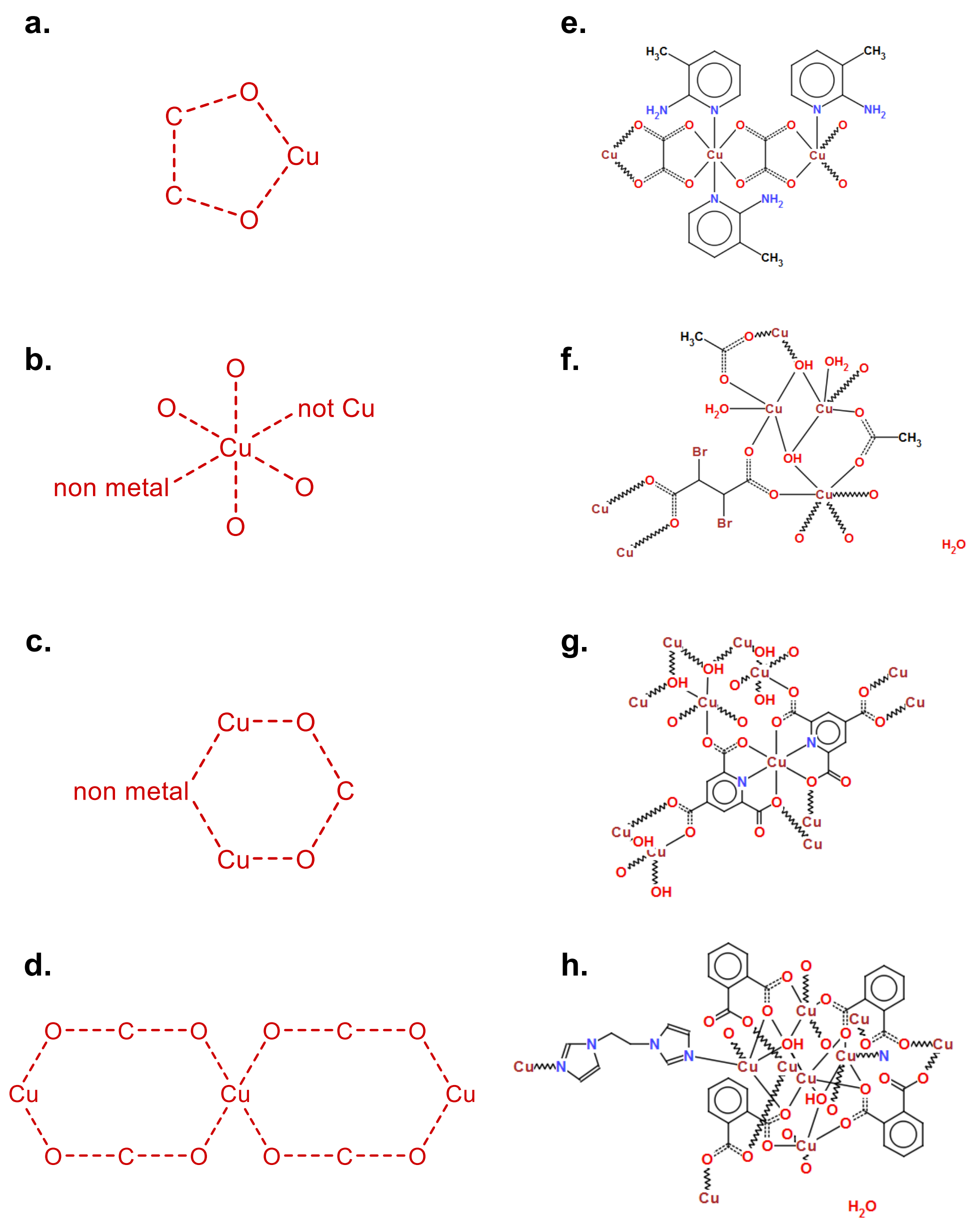

Figure S11. a. to d. Criteria used to eliminate undesired structures and the number of structures eliminated at each step. **a.** eliminates 128 hits, **b.** eliminates 190 hits, **c.** eliminates 88 hits, **d.** eliminates 5 hits. **e. to h.** Examples of eliminated structures corresponding to the criteria on the left. CSD refcodes: **e.** ABOCUP, **f.** AHEGIF, **g.** AGUMAR, **h.** ASEWEB.

MOF-74/CPO-27-type MOFs

A typical example of CPO-27 structure is shown in Figure S12. A similar approach as that of Zr-oxide-based MOFs was used here to find the target structures: Figure S13a represents the connection between the metal cluster and the organic linker. The metal atoms QA could be any of Zn, Cu, Ni, Co, Fe, Mn or Mg, which are, by comparison with a query where QA would simply be “any metal”, the most common metals in CPO-27-type of structures. This search leads to 147 hits. However, using only the criterion described in Figure S13a is not restrictive enough and a few undesired structures are found. An example is given in Figure S14. Adding a diagram which represents part of the ring to which the metal atoms belong to effectively eliminates 16 of these hits. Other untargeted structures are more difficult to remove; Figure S14 shows three “must not have” criteria that were developed based on specific examples, some of which are shown in the right column of Figure S14. The combination of all these criteria returns 108 hits.

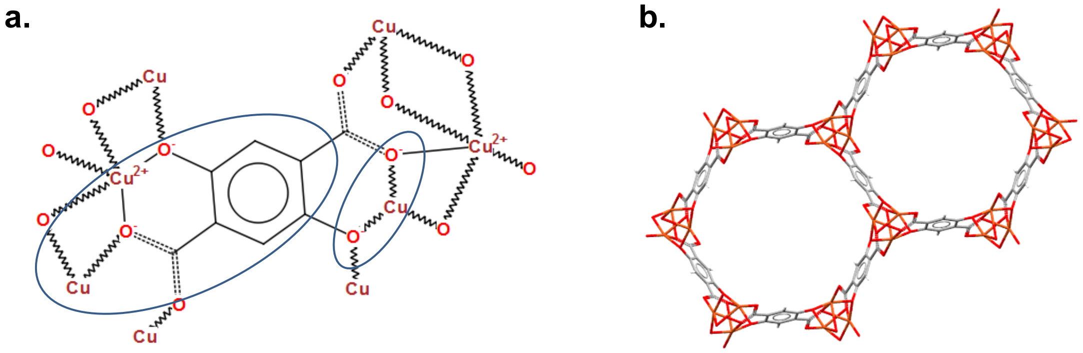

Figure S12. An example MOF-74/CPO-27 structure. **a.** Chemical diagram. The blue circled areas show the parts that are looked for by the search criterion in Figure S13. **b.** Spatial representation of the hexagonal channels formed in CPO-27. CSD refcode: COKNIB.

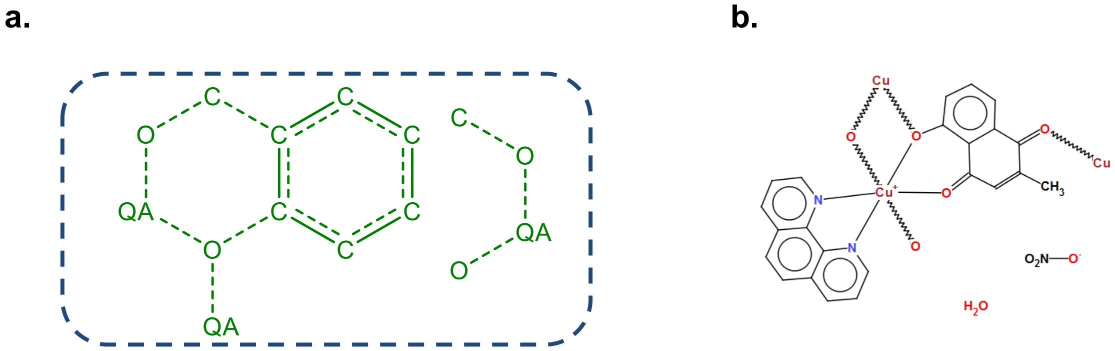

Figure S13. a. Criterion developed to look for MOF-74/CPO-27-type MOFs. Both parts should be considered as one single query. QA = Zn, Cu, Ni, Co, Fe, Mn, Mg. **b.** Example of an undesired structure found if the criterion is not restrictive enough, i.e. if only the left part of the criterion is represented. CSD refcode: XUVNUZ.

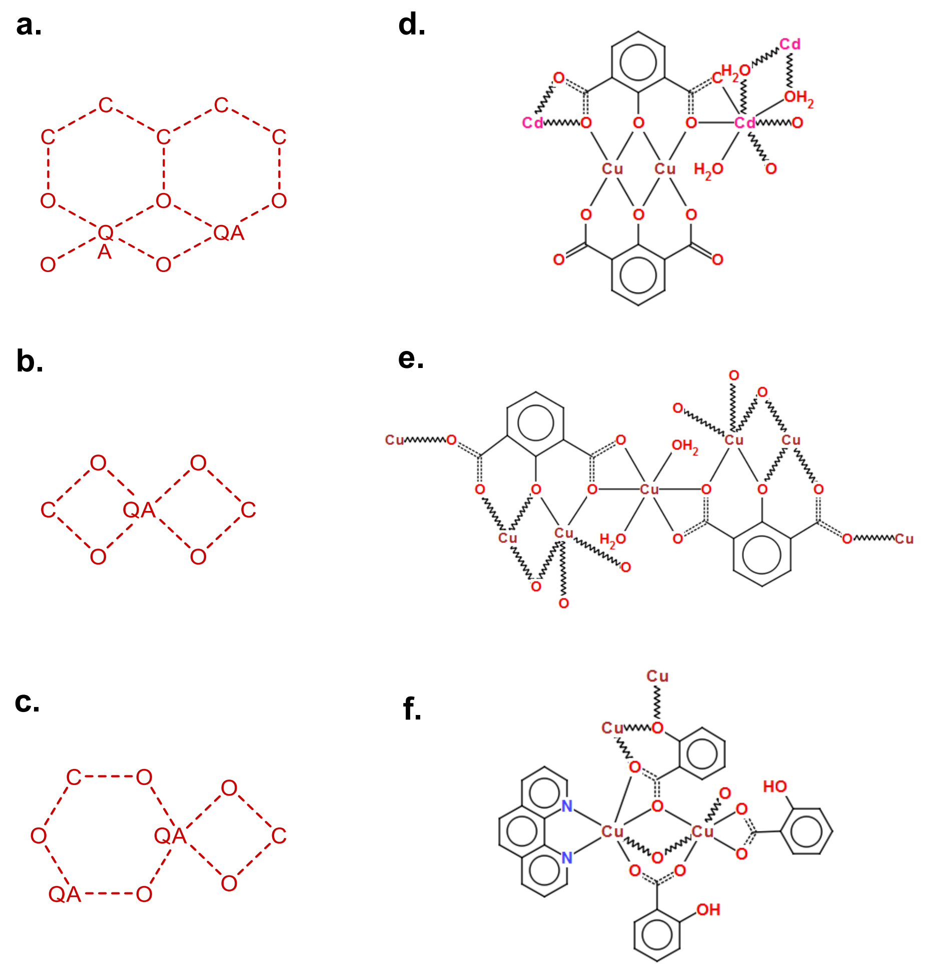

Figure S14. a. to c. Criteria developed to eliminate undesired structures. **a.** eliminates 20 hits, **b.** eliminates 1 hit and **c.** eliminates 2 hits. **d. to f.** Examples of structures eliminated with the corresponding criterion on the left. CSD refcodes: **d.** ADICIA, **e.** FODHIQ, **f.** WUYTUF.

ZIF-type MOFs:

In ZIF-type of structures, the metal is tetrahedrally coordinated with four imidazolates. A "must have" criterion was first developed by describing the connection between the metal atoms and the organic linker. One metal atom is linked to four nitrogen atoms, two of which are part of an imidazolates. For symmetry reasons, two imidazolates are enough. It is specifically stressed that the metal atom should only be bonded to four atoms (Figure S15). This search leads to 331 hits, of which some structures need to be removed. Figure S16 summarizes the list of "must not have" criteria. Diagrams a. to e. were developed based on specific structures, some of which are shown in the corresponding examples. A 3D "must not have" criterion was also added, as the cluster in some structures does not correspond to a tetrahedron, but are almost planar. A constraint with respect to the angle between two planes, each defined by a N-metal-N chain was added. As the data in the CSD are experimental, only a few clusters are close to a perfect tetrahedron. Therefore, different values of angles were tested in order to keep most of the tetrahedra, as deformed as they may be, and filter out clusters that are not tetrahedra. At the lower end, eliminating structures with an angle below 5° is not restrictive enough, and most flat clusters are included. At the higher end, eliminating structures with an angle below 30° is too restrictive. Though all the non-tetrahedra clusters were excluded, some flat tetrahedra were also filtered out. 25° was found to be a good cut-off value. Figure S16l shows an example of structure where the metal and the nitrogen atoms almost belong to the same plane. An additional criterion (Figure S17) used on its own targets ZIFs with a metal coordination number of 6 or 8.

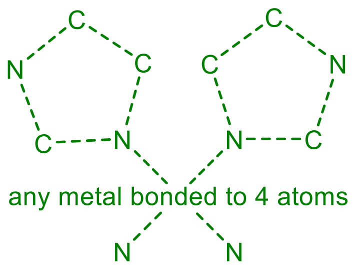

Figure S15. "Must have" criterion used to look for ZIF-type structures.

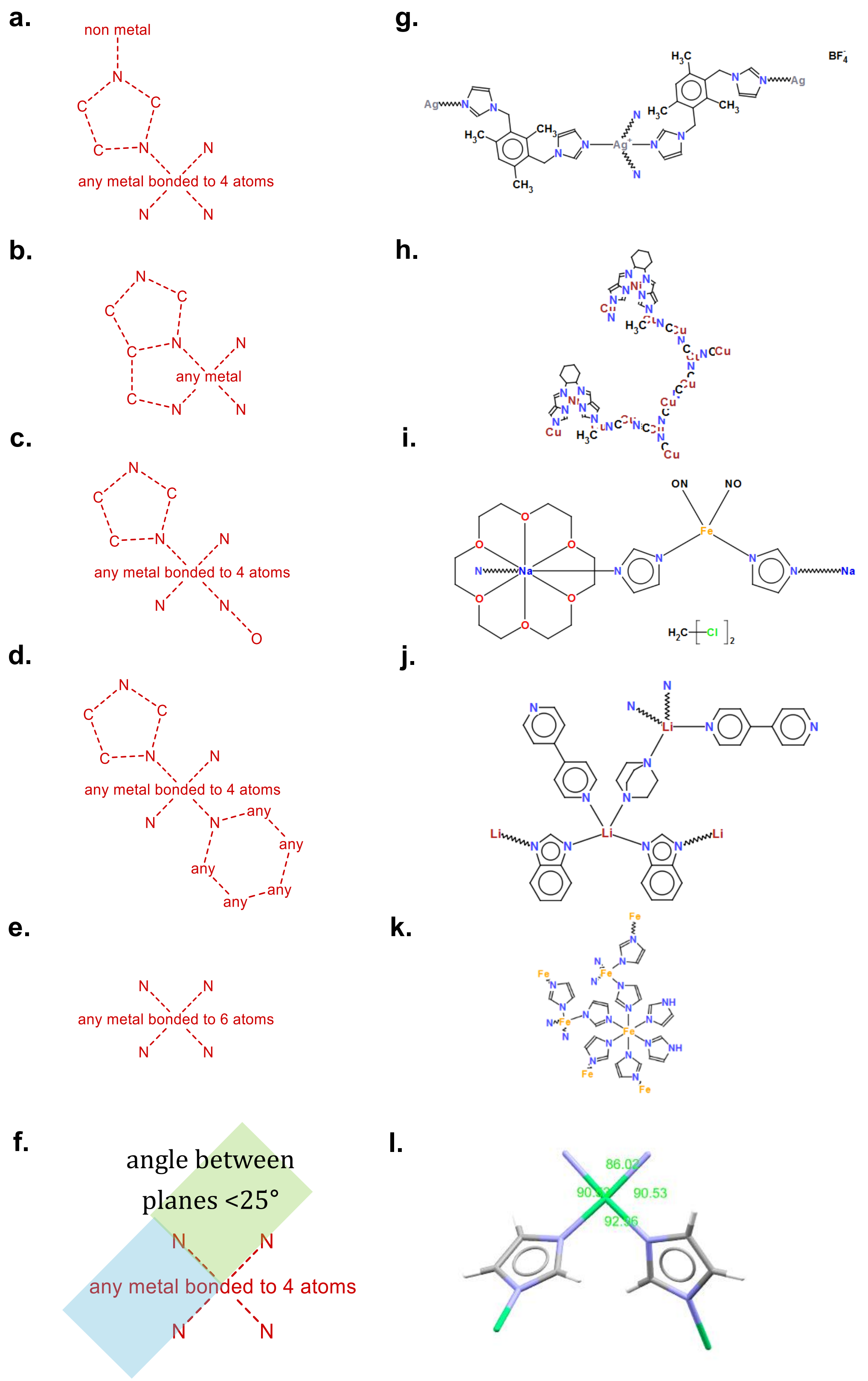

Figure S16. a. to f. "Must not have" criteria used to eliminate undesired structures. **a.** eliminates 99 hits, **b.** eliminates 4 hits, **c.** eliminates 1 hit, **d.** eliminates 6 hits, **e.** eliminates 7 hits, **f.** eliminates 4. **g. to l.** Example structures corresponding to the criterion described in the left. CSD refcodes: **g.** BUGKOF, **h.** KURPOE, **i.** VOBFIC, **j.** ALIHAF, **k.** IMIDFE, **l.** ALIDUU.

a. **b.**

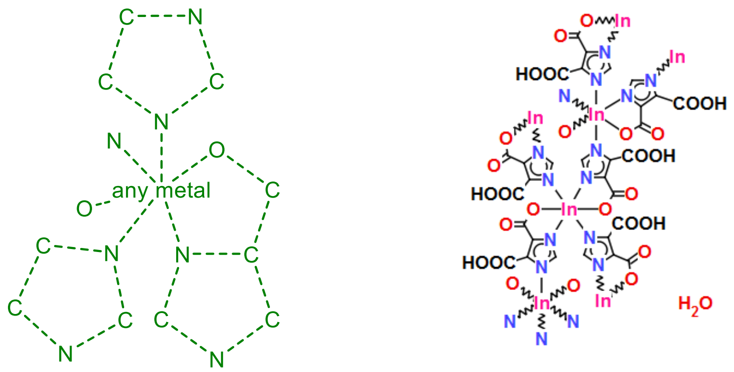

Figure S17. a. Criterion used to look for ZIF structures with metal coordination of 6 or 8. **b.** An example corresponding structure, CSD refcode: TEFWOR.

S3. MOFs containing functional groups

Halogen groups:

Figure S18 shows the criterion used to look for MOFs containing halogen groups. X represents any of F, Cl and Br and should be connected to only one other atom. X should not be part of the metal cluster, and should therefore not be bonded to a metal atom. In the particular case of F, the halogen atom should not be bonded to S or P. An example of undesired structure is given in Figure S19. The configuration of the bonds between the three non-metal atoms ensures that the functional group is attached to the organic linker, but is not part of the main chain of the linker. The variable bonds are either aromatic, delocalised, single or double, so that the functional groups that are looked for are not only those bonded to an aromatic structure, but also those that are linked to a linear organic chain. The second neighbors of the halogen atoms should not be halogens. An example of undesired structure for the -F group is given in Figure S20. A summary of the number of structures obtained for each case is given in Table S2.

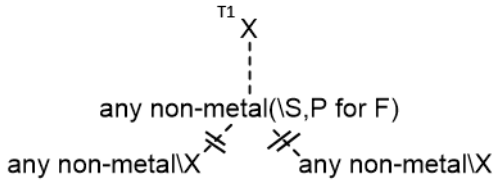

Figure S18. Criterion used to look for halogen groups. Replace X with F, Cl or Br. The variable bond is either single, double, aromatic or delocalized.

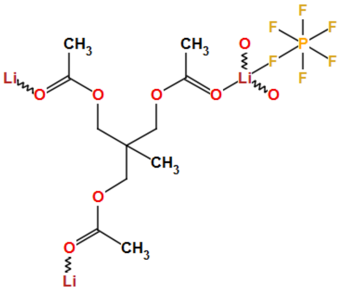

Figure S19. Example structures obtained for the -F group if the F atom is linked to a P atom. CSD refcode: WAHJOF.

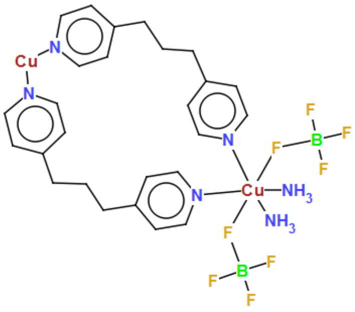

Figure S20. Example structure obtained for the -F groups if the second neighbors of the F atom are also F atoms. CSD refcode: ADOKOV.

Table S2. Number of structures obtained for each halogen group

Functional group	Number of hits
F	827
Cl	864
Br	503

The particular case of FMOFs:

Figure S21 shows the criterion used to find FMOFs in the MOF subset. It consists in describing the organic linker of the MOF. This search led to 12 structures.

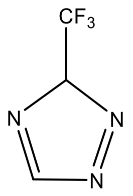

Figure S21. Criterion used to look for FMOFs.

Polar groups:

The criterion used for the polar groups is very similar to that of the halogen groups. Apart from -CN, the criterion shown in Figure S22 is enough to find structures containing -NH2, NO2, -COOH and -OH. It is also necessary to impose a number of bonded atoms to each atom of the polar groups. For instance, in -OH, O should be bonded to two atoms only, and H to only one.

For the particular case of -CN, an extra search was carried out in order to eliminate structures in which the cyanides are part of dicyanide functional groups. The criteria used to look for these dicyanides are shown in Figure S23. The list of structures obtained from this combination of searches is then eliminated from the main search presented in Figure S22.

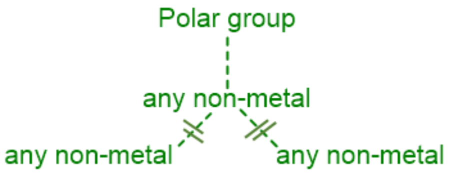

Figure S22. Criterion used to look for polar functional groups. The variable bond is either single, double, aromatic or delocalized.

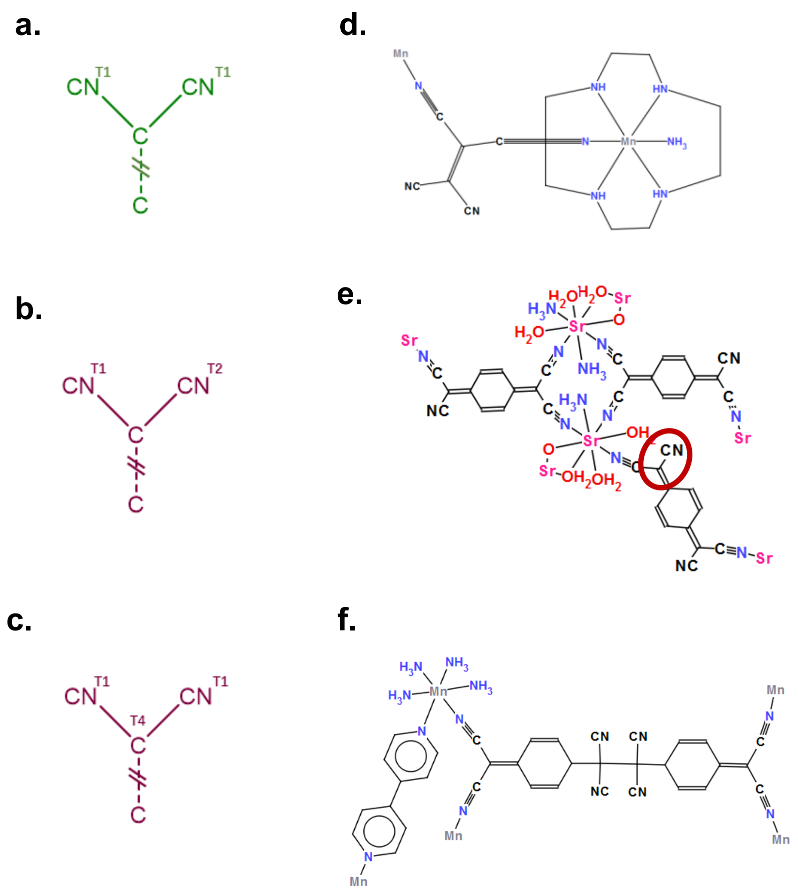

Figure S23. Criteria used to look for undesired dicyanide groups and corresponding examples for each criterion. The variable bonds are single or double. **a.** is the main criterion used to look for dicyanide functional groups. It is represented in green as it is a “must have” criterion for the search of dicyanides. **b.** is the criterion used to target dicyanide structures where one of the cyanide is part of the linker and the other cyanide can therefore be considered as a cyanide group. It is represented in red as it is a “must not have” criterion and returns structures that should be eliminated from the search for dicyanides. **c.** another “must not have” criterion used to look for structures where the carbon linked to the cyanide groups is bonded to more than three atoms. **d. to f.** Example structures for each case. CSD refcodes: **d.** AGAMUR, **e.** BENZOL, **f.** BUSQEM.

NB: the obtained list is to be eliminated from the main search corresponding to Figure S22, therefore the criteria in red are overall double negatives, i.e. positives.

Table S3. Number of hits obtained for each of the polar groups.

Functional group	Number of hits
NH_2	1996
NO_2	1198
COOH	1918
OH	1729
CN	520

Alkoxy groups:

A similar approach to that of polar groups leads to the criterion in Figure S24, where ‘alkoxy’ should be replaced by -OMe, -OEt and -OPr.

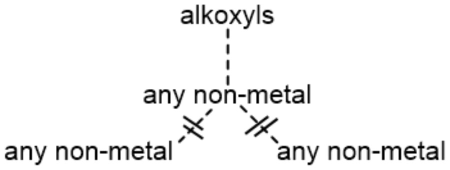

Figure S24. Criterion used to look for alkoxy groups. The variable bonds are either single, double, aromatic or delocalized.

Table S4. Number of hits for each alkoxy group.

Functional group	Number of hits
-OMe	707
-OEt	130
-OPr	31

Alkyl groups:

Using the same criterion previously described for alkoxy groups returns circa 20,000 structures for alkyl groups, of which about two thirds are not the target type. This is because alkyl groups are ubiquitous and are very often part of another functional group. One alternative way of looking for alkyl groups is to break down the search into three: one search (Figure S25a) looks for alkyl groups attached to aromatic structures *only*, another one looks for alkyl groups attached to a linear chain, and more specifically via single bonds, the last one looks for groups attached a linear chain via a single bond and a double bond, or groups attached to an aromatic ring represented with single and double bonds.

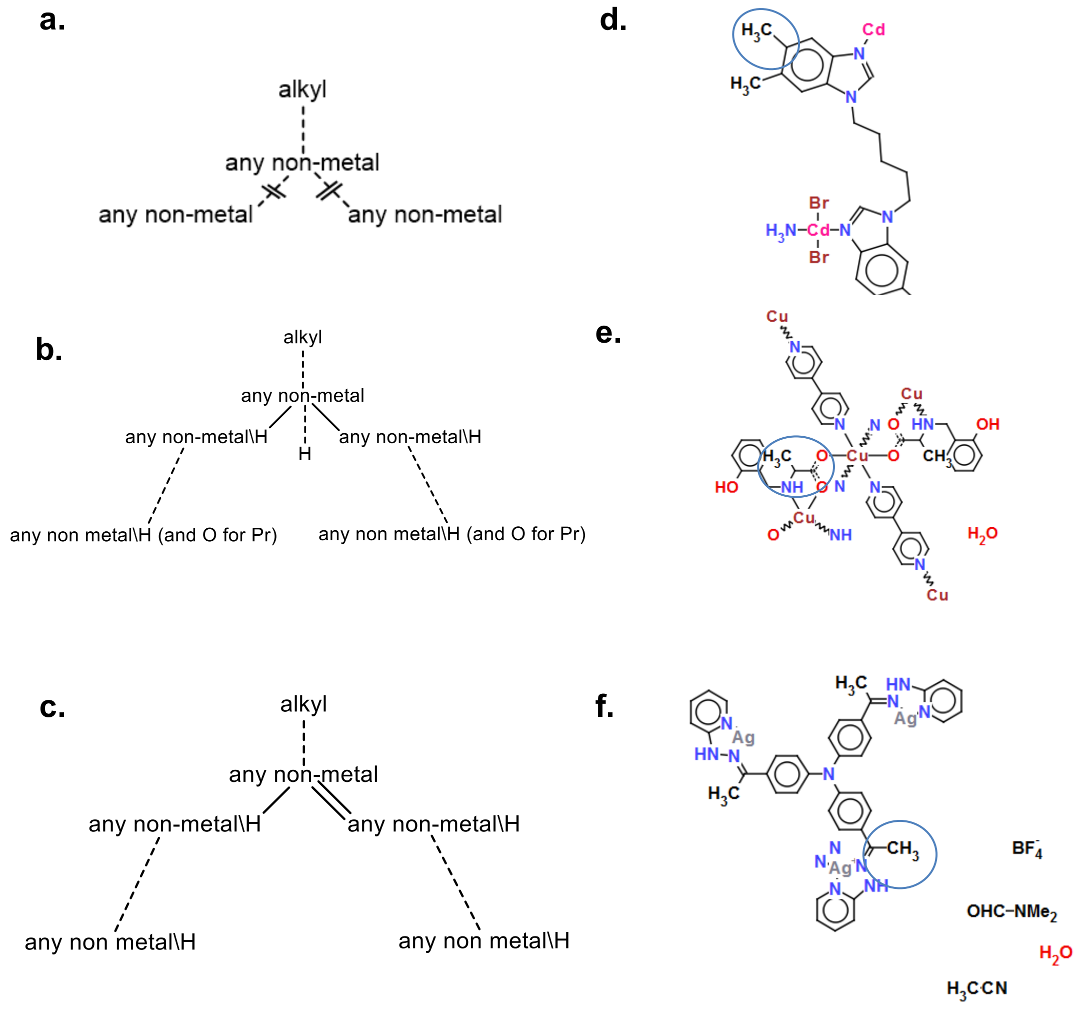

Figure S25. a. to c. Criteria used to look for structures with alkyl functional groups. The variable bonds in **a.** are aromatic or delocalized. **d. to f.** Example structures targeted by each criterion. The circles highlight the area captured by each criterion. CSD refcodes: **d.** ACELAX, **e.** ACABEM, **f.** ADAVEI.

Table S5. Number of hits for each alkyl group

Functional group	Number of hits
Me	7126
Et	437
Pr	126

Alkyl groups with more than 4 carbon atoms

For alkyl groups with more than 4 carbon atoms, the combination of criteria in Figure S26 can be used. The two green "must have" criteria each describe one end of the alkane chain: it is bonded to the linker on one side and ends with -CH_3 on the other. They are grouped together to form an "AND" statement: each structure should meet both criteria. The resulting hitlist contained undesired structures which are eliminated using the red "must not have" criterion. The final hitlist contains 261 structures.

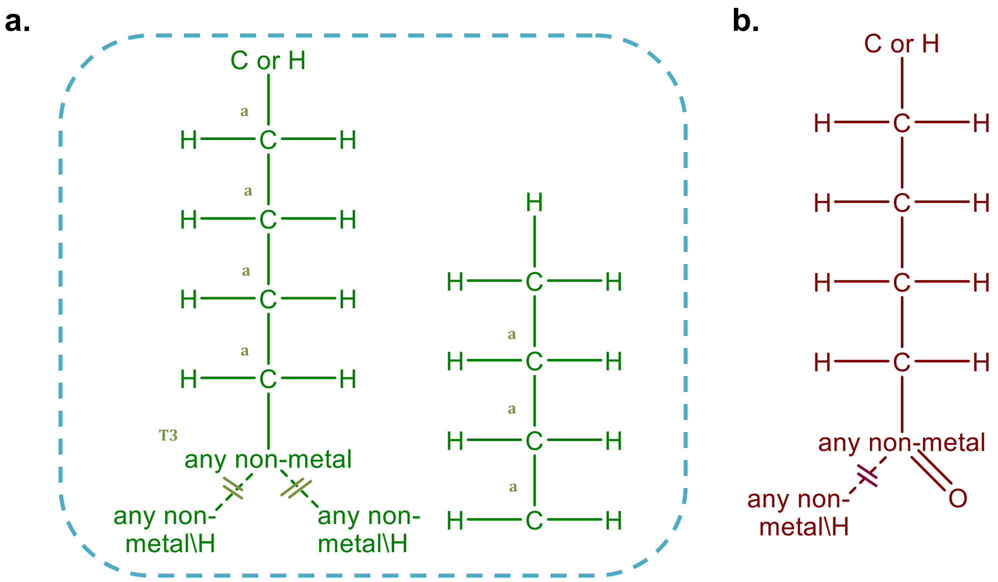

Figure S26. Criteria used to look for alkyl groups of more than 4 carbon atoms. **a.** constraints on both ends of the chain: one end has to be bonded to the linker but not be part of it and the other must be free and end with -Me. **b.** eliminates undesired structures. The variable bonds are either single, double, aromatic or delocalized. Upperscript a: the corresponding atom is acyclic. T3: the corresponding atom can only be bonded to three other atoms.

Perfluoroalkane groups

Another set of criteria is used to target structures with perfluoroalkane groups. It follows the same reasoning as above: each of the two green "must have" queries describe one end of the chain and are grouped together to form an "AND" statement, and the red "must not have" criterion eliminates undesired structures. The hitlist contains 64 structures.

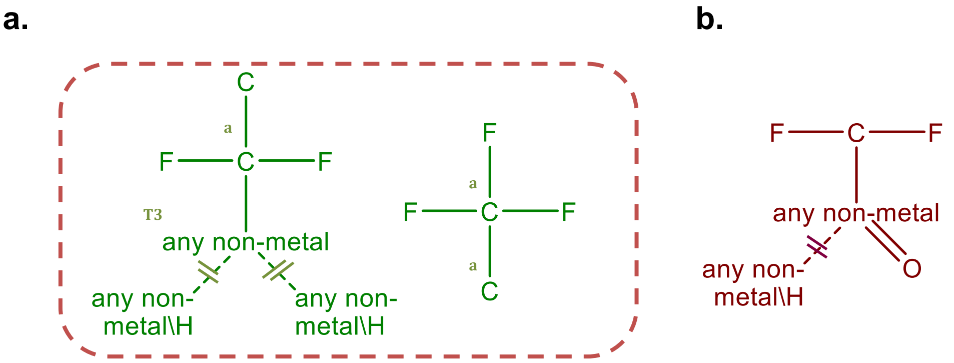

Figure S27. Criteria used to look for structures with perfluoroalkane chains. **a.** constraints on both ends of the perfluoroalkane group: one end is bonded to a linker but not part of it, the other must end with -CF_3. **b.** eliminates undesired structures. The variable bonds are either single, double, aromatic or delocalized. Superscript a: the corresponding atom is acyclic.

S4. Calculations of MOF physical and geometrical properties, dimensionalities of frameworks and channels

Zeo++ software package[2] was used to characterize and calculate the geometric properties for all cleaned MOF structures (i.e. after removing solvent molecules). The calculations are based on Voronoi network generation[2] and analyzing it for geometrical parameters such as the largest cavity diameter (LCD) and the pore limiting diameter (PLD). The calculations of accessible surface area and pore volume were performed by using a spherical N_2 probe with a radius of 1.86 Å, whilst geometrical volume fraction was calculated by setting the radius of the probe to zero. N_2 probe was also used to determine the dimensionality of framework channels. Structural dimensionality for each framework was also determined from atom connectivity matrix in Zeo++. Covalent radii from the CSD were used for all MOF atoms.

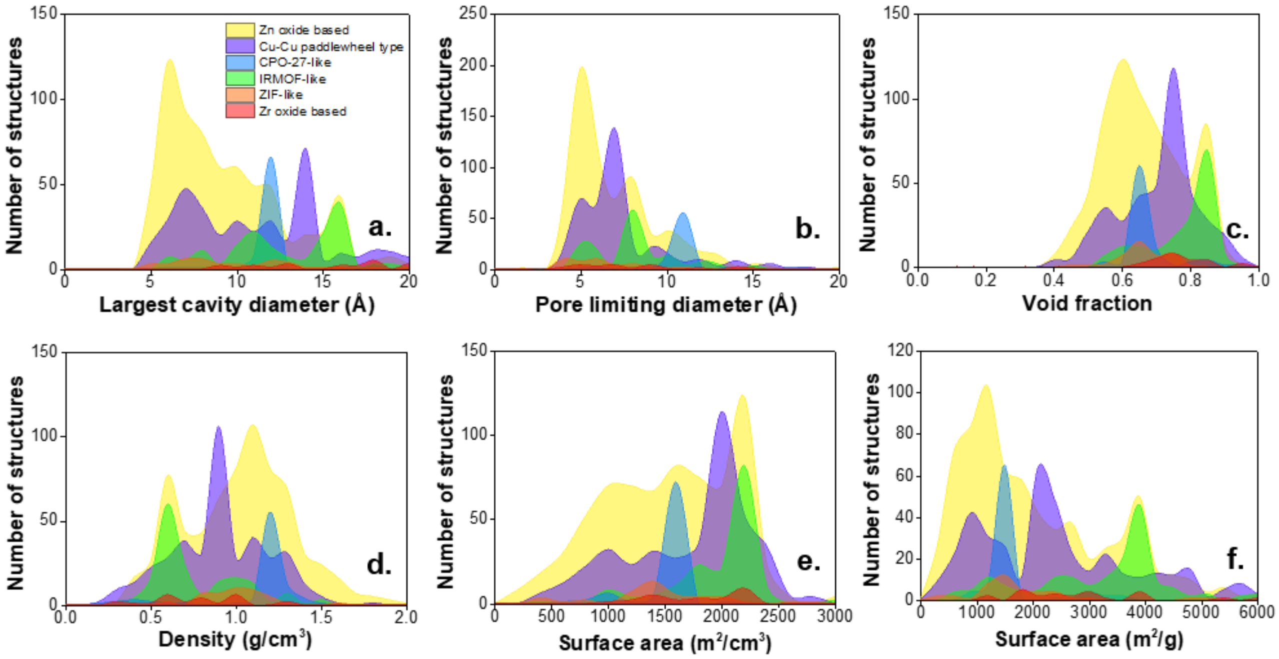

Figure S28. Histograms showing the geometric properties for each MOF family identified in the CSD MOF subset. **a.** Largest cavity diameter, **b.** pore limiting diameter, **c.** void fraction, **d.** density, **e.** gravimetric surface area, **f.** volumetric surface area.

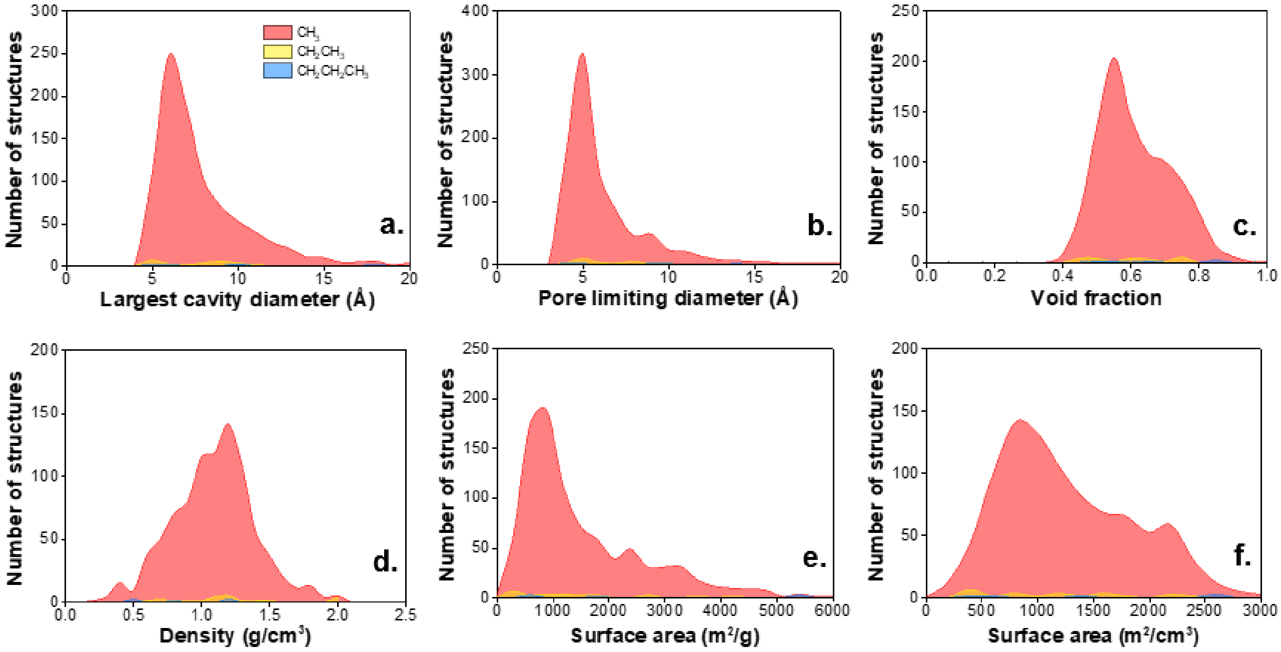

Figure S29. Histograms showing the number of hits for MOFs with different alkyl groups. **a.** Largest cavity diameter, **b.** pore limiting diameter, **c.** void fraction, **d.** density, **e.** gravimetric surface area, **f.** volumetric surface area.

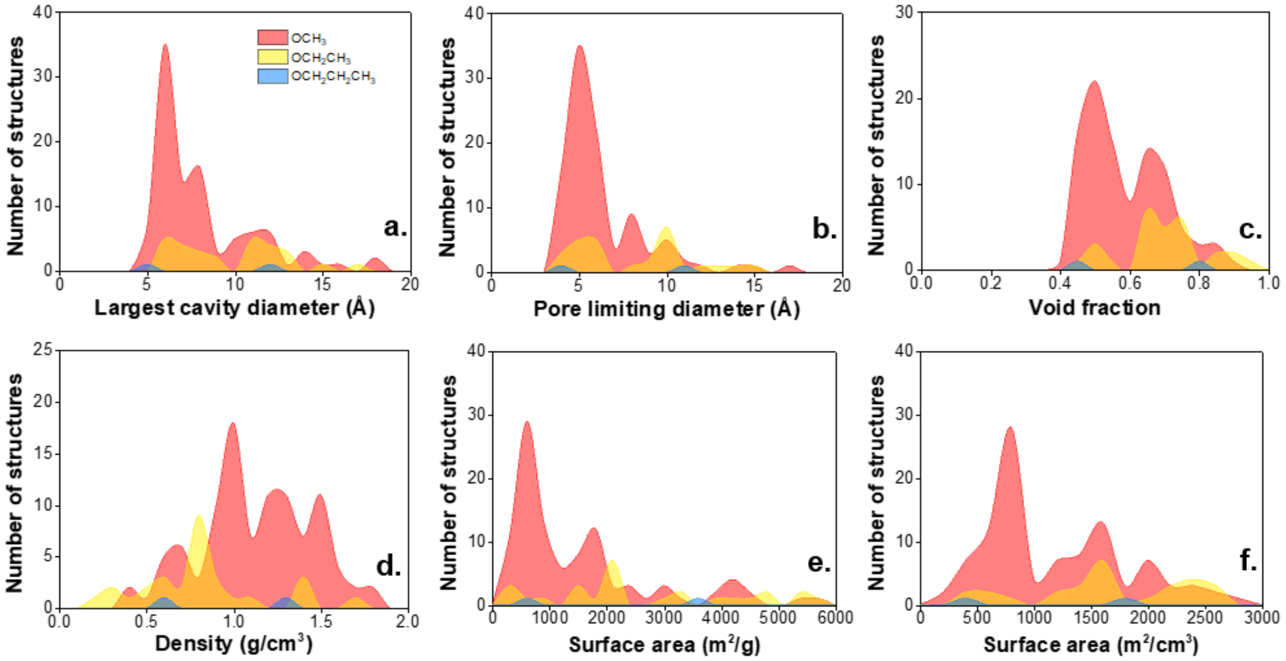

Figure S30. Histograms showing the number of hits in MOFs with different alkoxy groups. **a.** Largest cavity diameter, **b.** pore limiting diameter, **c.** void fraction, **d.** density, **e.** gravimetric surface area, **f.** volumetric surface area.

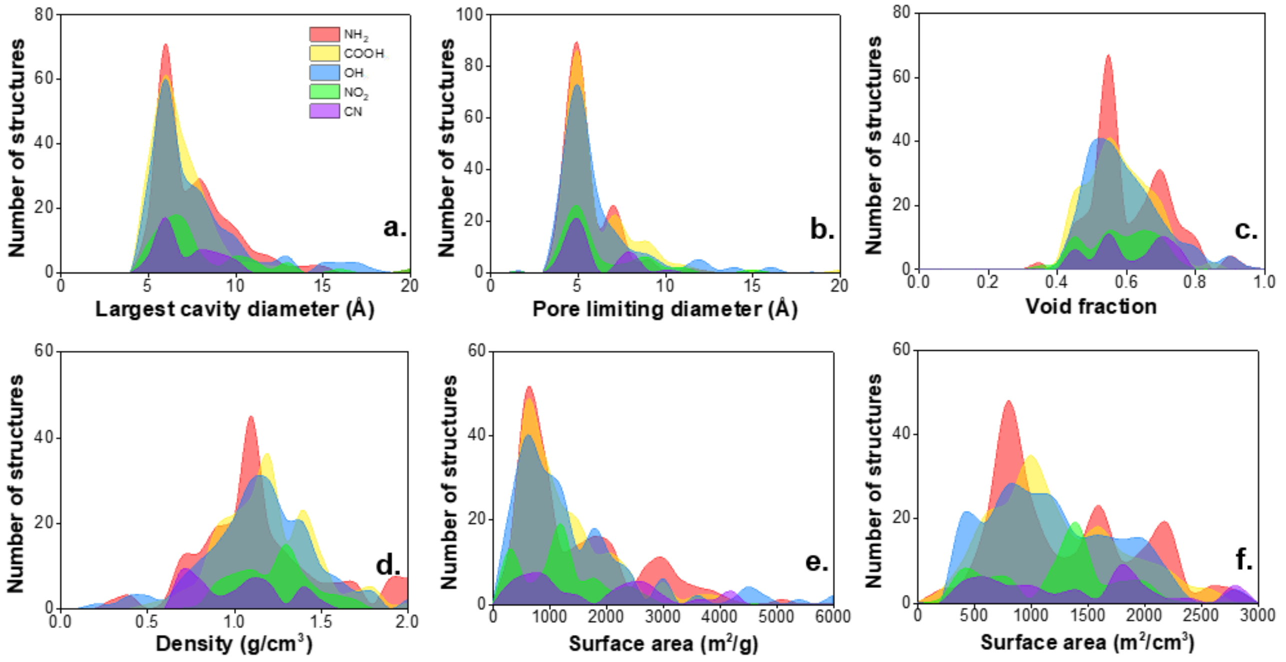

Figure S31. Histograms showing the number of hits in MOFs with different polar groups. **a.** Largest cavity diameter, **b.** pore limiting diameter, **c.** void fraction, **d.** density, **e.** gravimetric surface area, **f.** volumetric surface area.

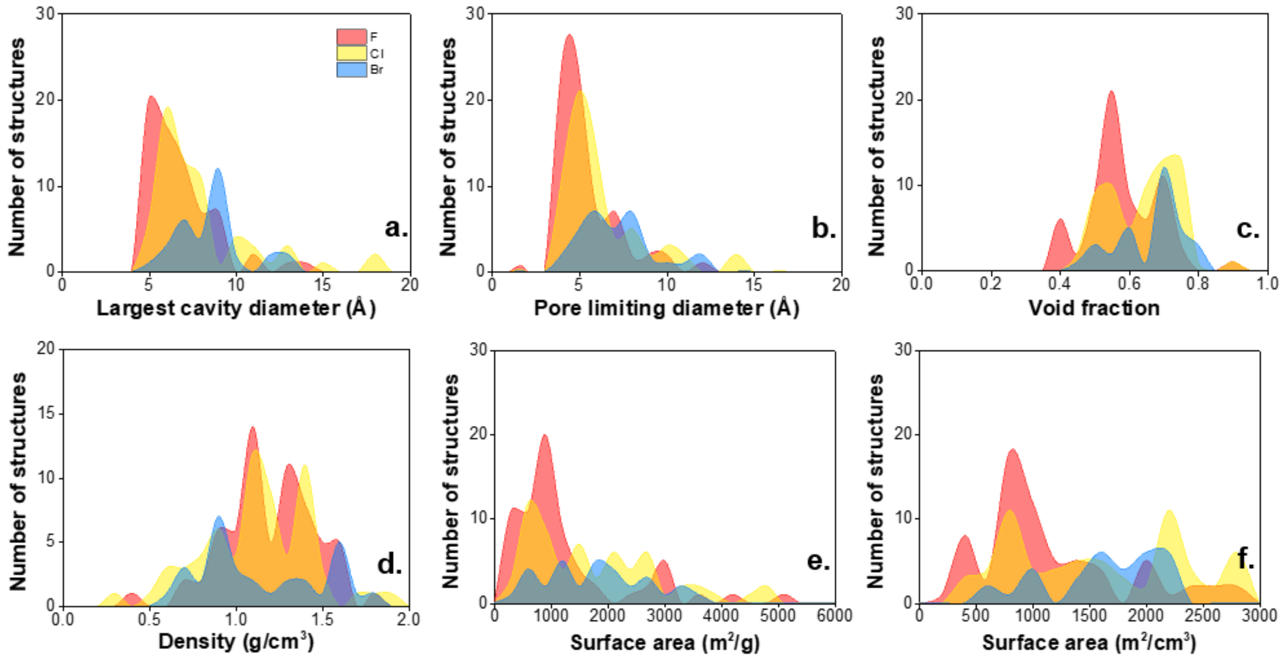

Figure S32. Histograms showing the number of hits in MOFs with different halogen groups. **a.** Largest cavity diameter, **b.** pore limiting diameter, **c.** void fraction, **d.** density, **e.** gravimetric surface area, **f.** volumetric surface area.

The calculation of the framework dimensionalities was performed with our in-house CSD Python API script, available in the SI. The channel dimensionalities were obtained using PoreBlazer, as explained in the manuscript.

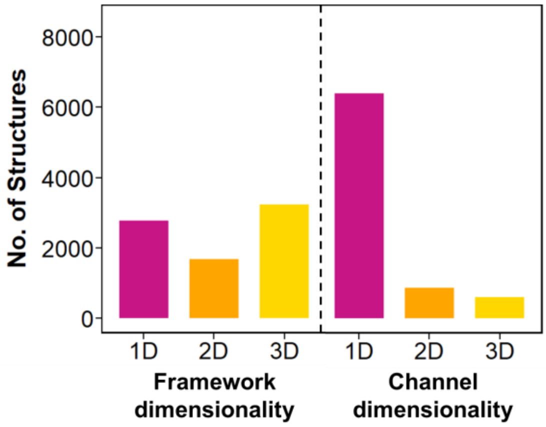

Figure S33. Histograms of framework and channel/pore dimensionalities for 8,253 porous MOFs. The channel dimensionalities of some structures could not be determined and are therefore not shown.

S5. Quality assessment of the data in the MOF subset using R factors

The R-factors and crystal systems were extracted using the CSD Python API. The geometric and physical properties were obtained using Zeo++, as explained above.

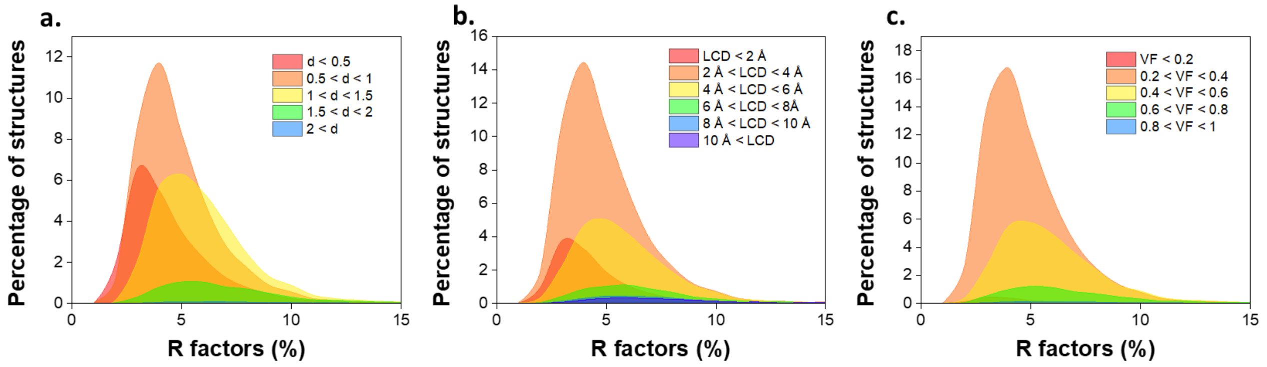

Figure S34. Histograms of **a.** density (g/cm^3), **b.** largest cavity diameter (LCD) and **c.** void fraction against R factors for structures with non-zero gravimetric surface area values in the CSD MOF subset.

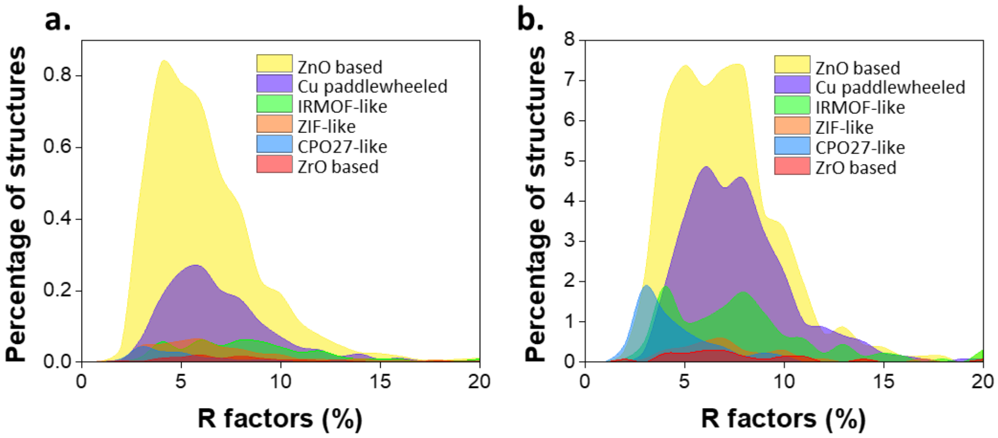

Figure S35. a. Histograms of R-factors for the different MOF families. **b.** Histograms of R-factors for the different MOF families with non-zero surface area.

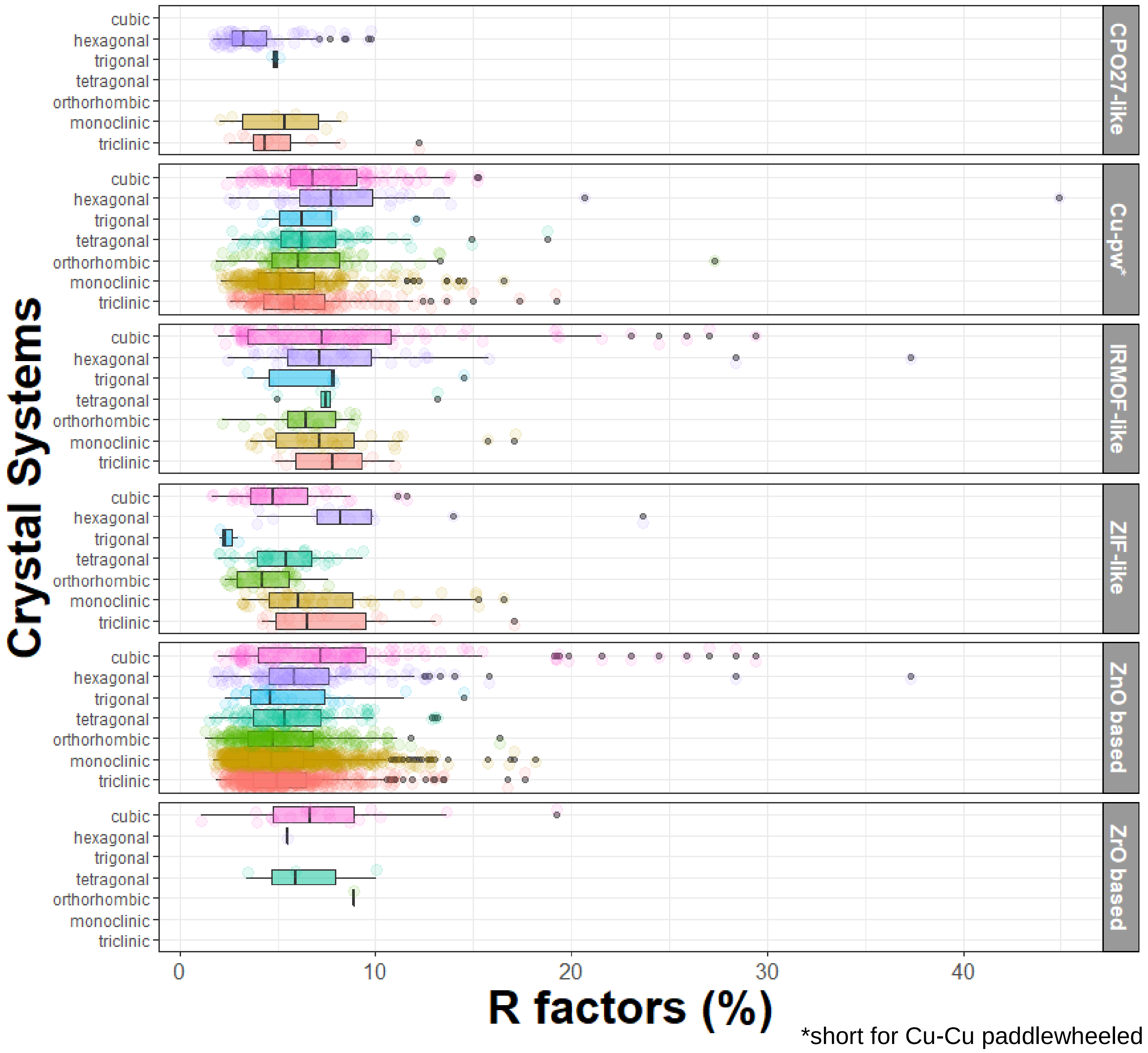

Figure S36. Boxplots of R factors vs. crystal systems for each MOF family.

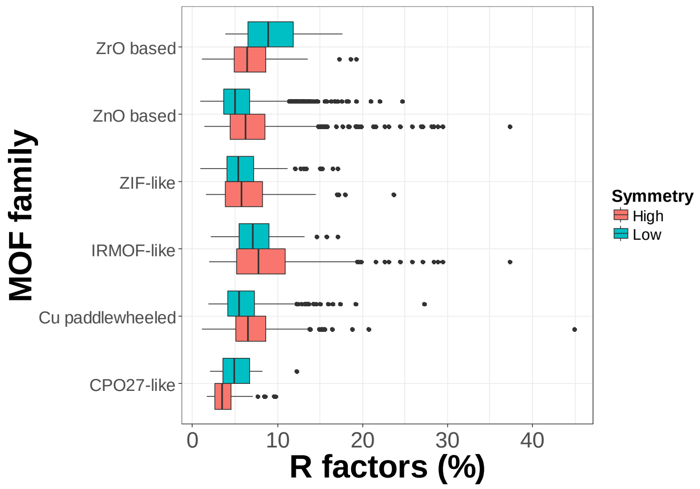

Figure S37. Boxplots of R factors vs. degree of symmetry for of their crystal systems for each MOF family.

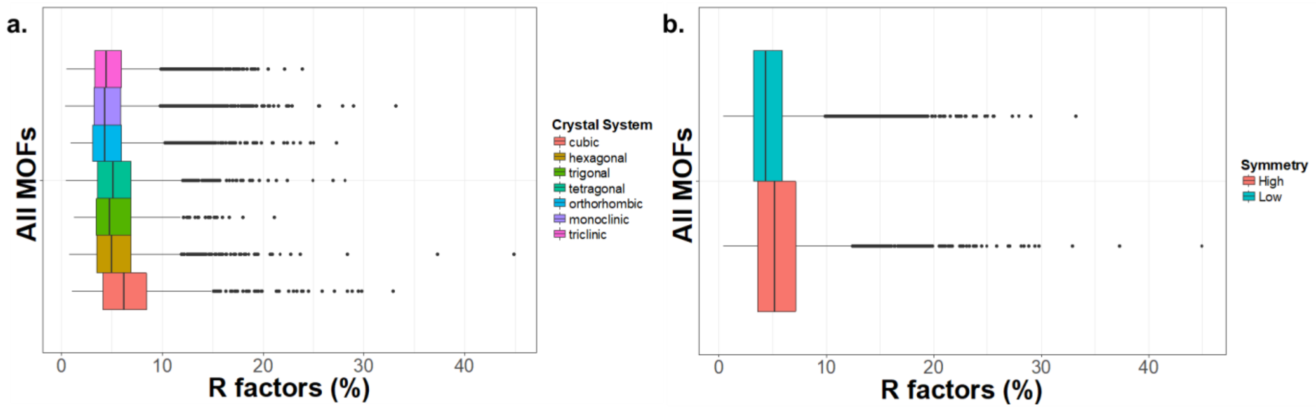

Figure S38. Boxplots of R factors vs. **a.** crystal systems and **b.** degree of symmetry for all structures in the CSD MOF subset.

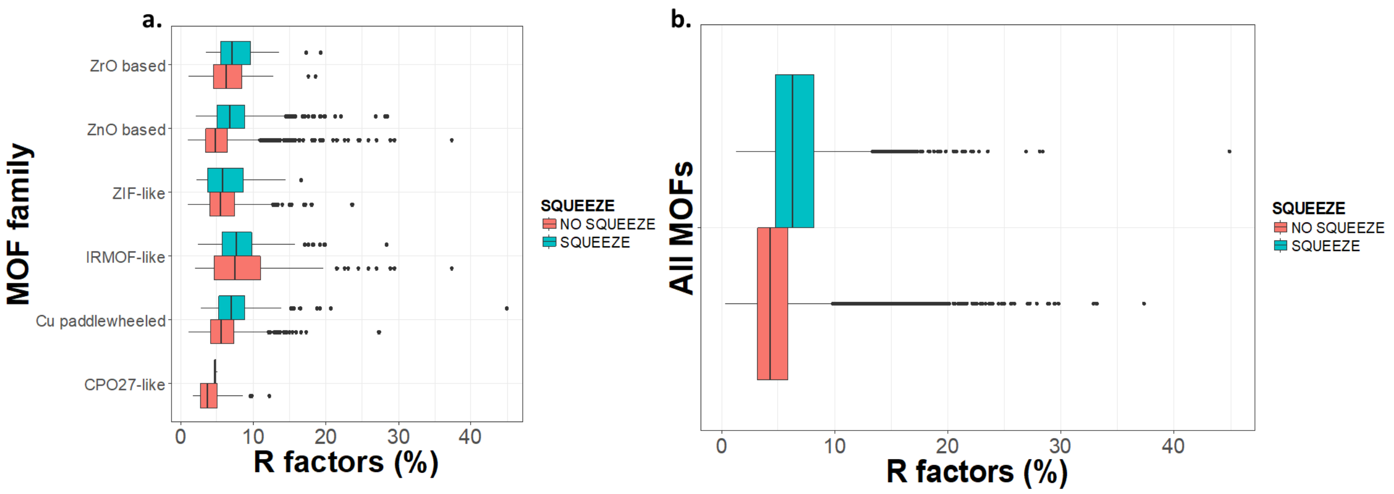

Figure S39. Boxplots of R factors for **a.** different MOF families and **b.** all structures in the CSD MOF subset. Only 4,769 structures from the MOF subset have gone through the SQUEEZE process.

S6. GCMC simulations

Table S6. Force field parameters used in the GCMC simulations. Raspa's force field file is also included in the supplementary information.

	ε (K)	σ (Å)
B_	47.804	3.582
C_	47.854	3.474
H_	7.649	2.847
N_	38.948	3.263
O_	48.156	3.034
P_	161.024	3.698
S_	173.101	3.591
F_	36.482	3.094
I_	256.632	3.698
K_	17.612	3.397
V_	8.051	2.801
W_	33.714	2.735
Y_	36.23	2.981
U_	11.07	3.025
Li_	12.58	2.184
Be_	42.772	2.446
Al_	155.992	3.912
Si_	155.992	3.805
Cl_	142.557	3.52
Na_	251.6	2.801
Mg_	55.855	2.692
Ga_	201.28	3.912
Ge_	201.28	3.805
As_	206.312	3.698
Se_	216.376	3.591
Br_	186.184	3.52
Ca_	25.16	3.094
Sc_	9.561	2.936
Ti_	8.554	2.829
Cr_	7.548	2.694
Mn_	6.542	2.638
Fe_	27.676	4.045
Co_	7.045	2.559
Ni_	7.548	2.525
Cu_	2.516	3.114
Zn_	27.676	4.045
In_	276.76	4.09
Sn_	276.76	3.983
Sb_	276.76	3.876
Te_	286.824	3.769
Rb_	20.128	3.666
Sr_	118.252	3.244

Zr_	34.721	2.784
Nb_	29.689	2.82
Mo_	28.179	2.72
Tc_	24.154	2.671
Ru_	28.179	2.64
Rh_	26.67	2.61
Pd_	24.154	2.583
Ag_	18.115	2.805
Cd_	114.73	2.538
Tl_	342.176	3.873
Pb_	333.622	3.829
Bi_	260.658	3.894
Po_	163.54	4.196
At_	142.909	4.233
Rn_	124.794	4.246
Cs_	22.644	4.025
Ba_	183.165	3.3
Hf_	36.23	2.799
Ta_	40.759	2.825
Re_	33.211	2.632
Os_	18.618	2.78
Ir_	36.734	2.531
Pt_	40.256	2.454
Au_	19.625	2.934
Hg_	193.732	2.41
Fr_	25.16	4.366
Ra_	203.293	3.276
La_	8.554	3.138
Ce_	6.542	3.169
Pr_	5.032	3.213
Nd_	5.032	3.186
Pm_	4.529	3.161
Sm_	4.026	3.137
Eu_	4.026	3.112
Gd_	4.529	3.001
Tb_	3.522	3.075
Dy_	3.522	3.055
Ho_	3.522	3.038
Er_	3.522	3.022
Tm_	3.019	3.006
Yb_	114.73	2.99
Lu_	20.631	3.243
Ac_	16.606	3.099
Th_	13.083	3.026
Pa_	11.07	3.051
Np_	9.561	3.051
Pu_	8.051	3.051
Am_	7.045	3.013

Cm_	6.542	2.964
Bk_	6.542	2.975
Cf_	6.542	2.952
Es_	6.038	2.94
Fm_	6.038	2.928
Md_	5.535	2.917
No_	5.535	2.894
Lw_	5.535	2.883
CH4_sp3	148	3.73
CH3_sp3	98	3.75
CH2_sp3	46	3.95
CH_sp3	10	4.65
C_sp3	0.5	6.4
CH2_sp2	85	3.675
CH_sp2	47	3.73
C_sp2	20	3.85
CH4_sp3dub	158.5	3.72
CH3_sp3dub	108	3.76
CH2_sp3dub	56	3.96
CH3_sp3gss	108	3.76
CH_sp2gss	51	4
CH2_sp2gss	93	3.72
CH2_sp2bae	83	3.72
CH2_sp2fco	93	3.685
dummy_bae	none	
dummy_fco	none	
com_gss	none	
H_com	36.7	2.958
C_co2	27	2.8
O_co2	79	3.05
N_n2	36	3.31
N_com	none	
N_nh3	185	3.42
H_nh3	none	
O_o2	49	3.02
O_com	none	
C_co	16.141	3.636
O_co	98.014	2.979
COM_co	none	
C_benz	30.7	3.6
H_benz	25.45	2.36
N_dmf	80	3.2
Co_dmf	50	3.7
Cm_dmf	80	3.8
O_dmf	100	2.96
H_dmf	8	2.2
C_aro	35.24	3.55
H_aro	15.08	2.42

CH3_aro	85.47	3.8
Ow	78	3.15365
Ar	119.8	3.34
Kr	166.4	3.636
Xe	221	4.1
He	10.9	2.64
Ne	35.7	2.789
H2	34.2	2.96

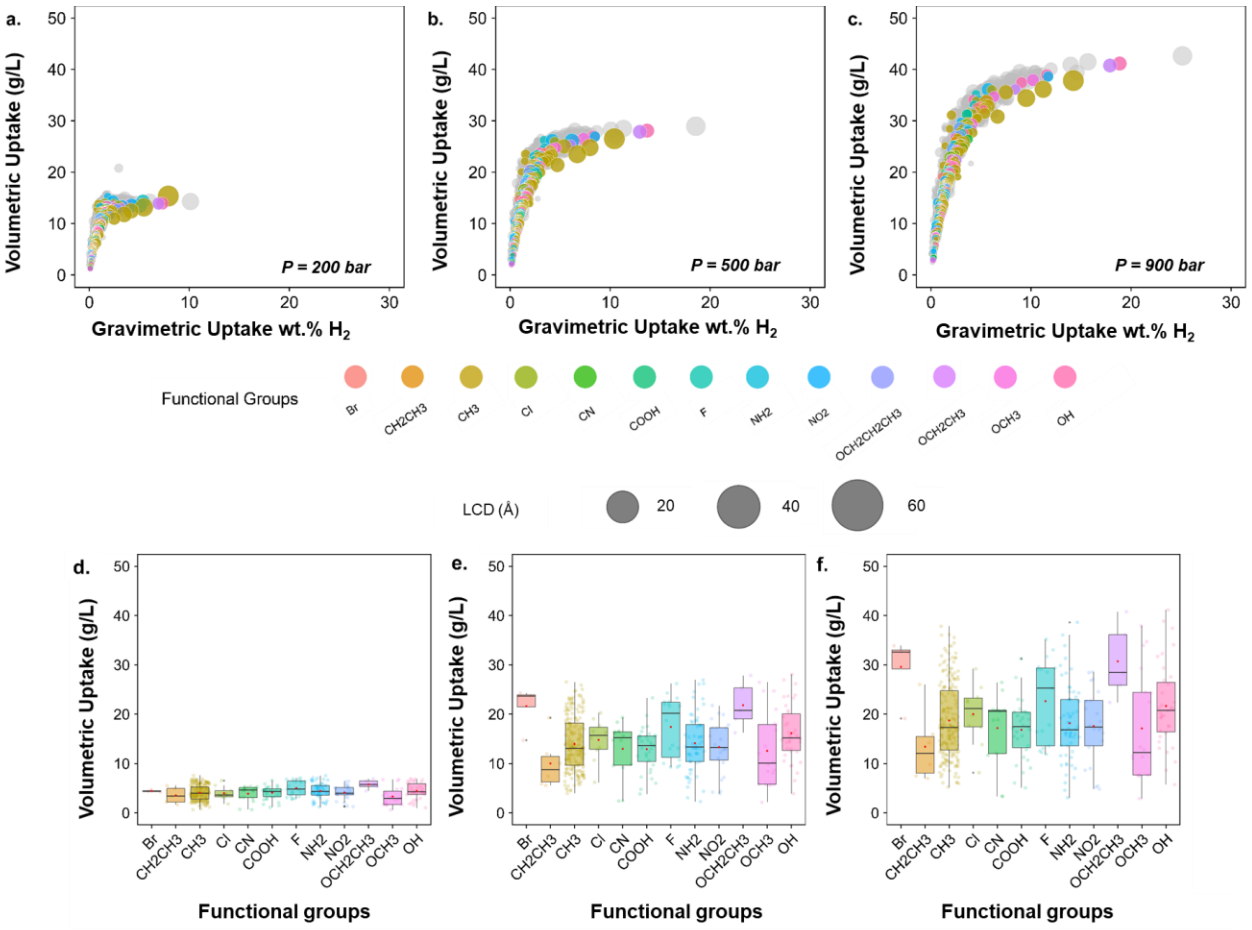

Figure S40. a.-c. Volumetric uptake versus gravimetric uptake in wt.% H_2 for the screened structures for hydrogen storage at **a.** 200 bars, **b.** 500 bars and **c.** 900 bars. Each circle corresponds to a structure. The colors highlight the functional groups the structures contain, the size of the circles indicate the LCD. **d.-f.** Boxplots of the volumetric uptake for each functional group. The markers represent the minimum, first quartile, median, third quartile, and maximum values, respectively. Outliers are represented by black data points.

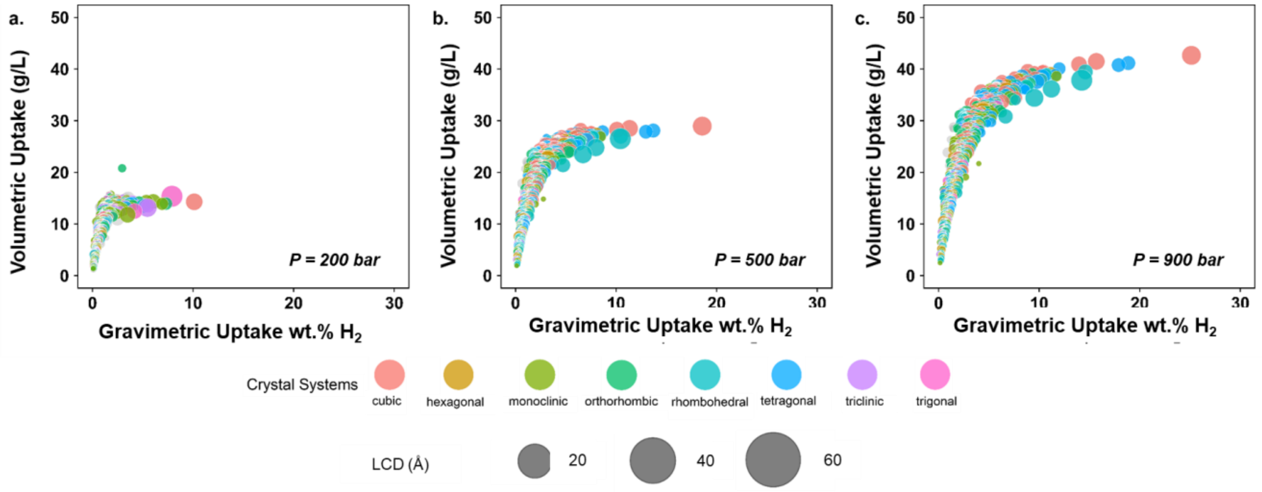

Figure S41. Volumetric uptake versus gravimetric uptake in w%H_2 for the screened structures for hydrogen storage at **a.** 200 bars, **b.** 500 bars and **c.** 900 bars. Each circle corresponds to a structure. The colors highlight the crystal systems of each structure, and the size of the circles indicate the LCD.

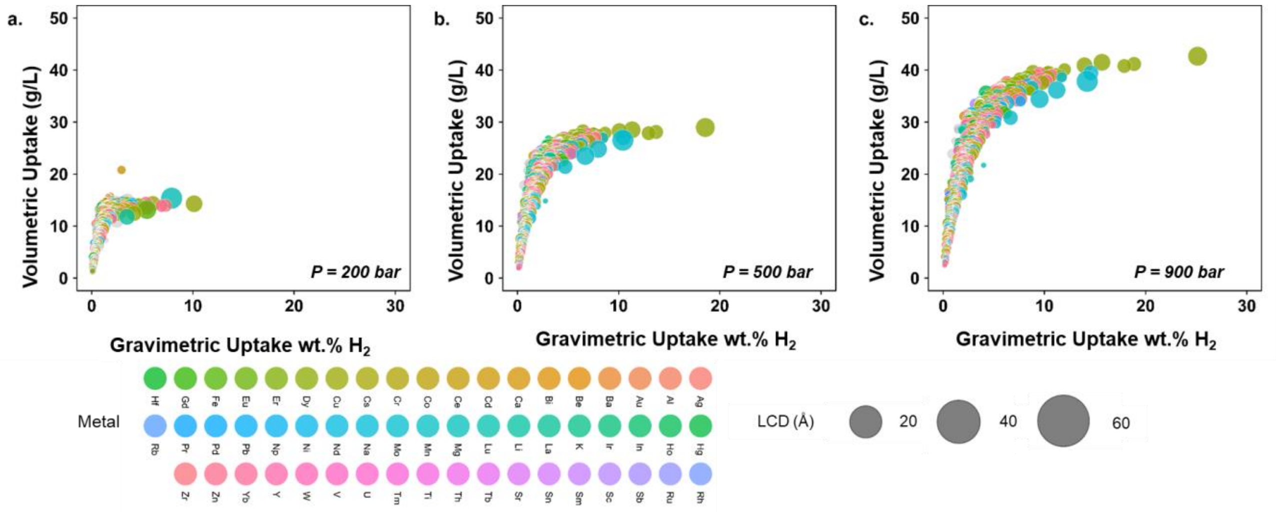

Figure S42. Volumetric uptake versus gravimetric uptake in w%H_2 for the screened structures for hydrogen storage at **a.** 200 bars, **b.** 500 bars and **c.** 900 bars. Each circle corresponds to a structure. The colors highlight a metal present in each structure, and the size of the circles indicate the LCD.

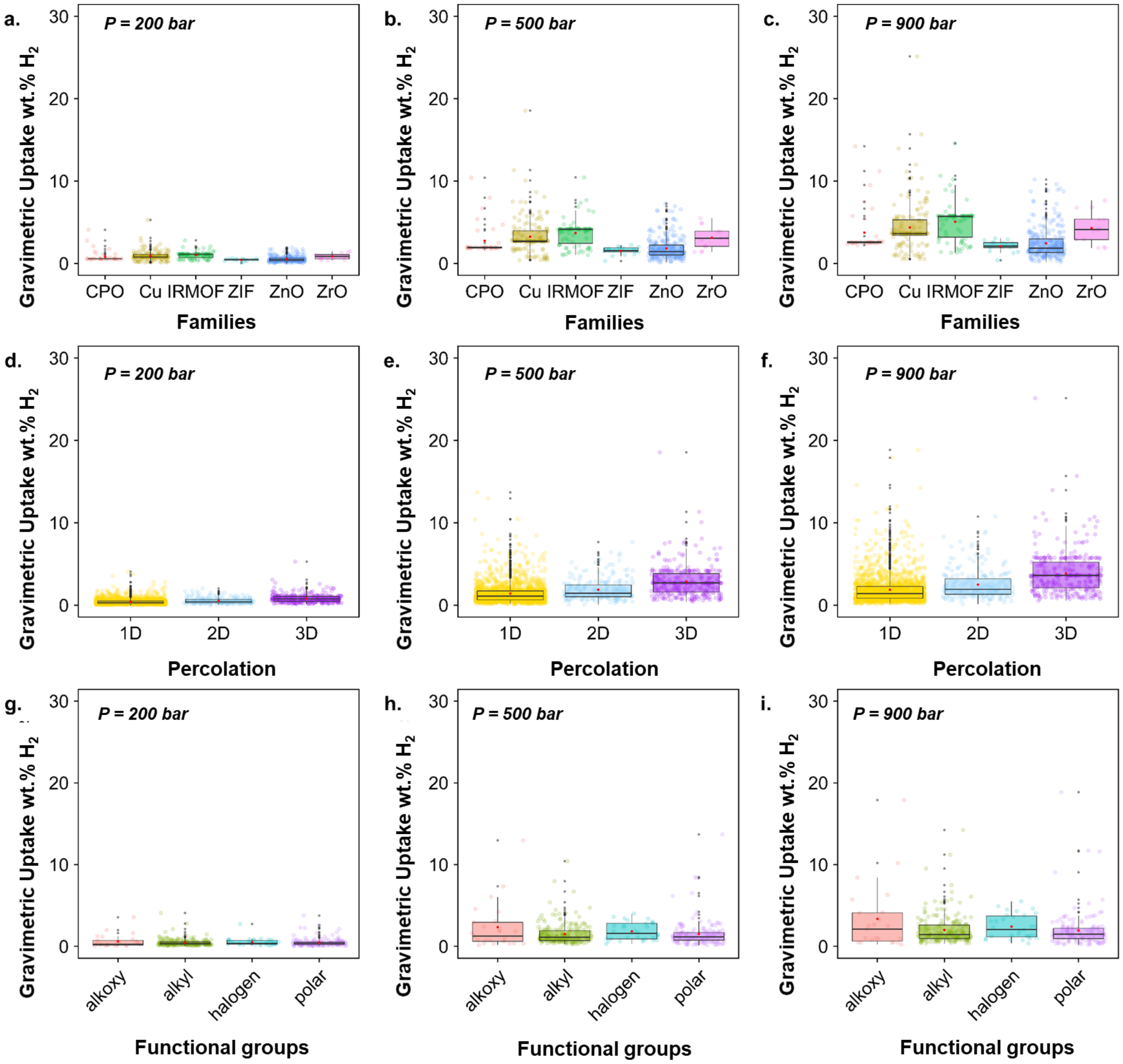

Figure S43. Quantitative characterization of the 3D MOFs screened for hydrogen storage. Boxplots of gravimetric uptake of H_2 at room temperature at 200, 500 and 900 bars versus **a.-c.** families of the screened structures, **d.-f.** percolation of the screened structures and **g.-i.** functional groups identified in the screened structures. The jittered points in the background give an idea on the number of structures considered for each boxplot. The markers represent the minimum, first quartile, median, third quartile, and maximum values, respectively. Outliers are represented by black data points.

S7. Database adjustment and general guidelines and updates

Presence of a few non-MOF structures

280 non-MOF structures have been found in the MOF subset (representing 0.003% of the subset). These structures usually contain several substructures, of which some are single molecules while others are 'polymeric' (i.e. can be extended to form a framework). The previously defined criteria aimed to look for patterns only in the polymeric part of the structure. However, in these cases, they wrongly targeted the single molecules. This anomaly has been rectified by using an additional check with a new feature from the CSD Python API component.is_polymeric (see below).

Updated function add_hydrogen

In a similar manner to above, the add_hydrogen function relied upon the correct identification of polymeric components in order to correctly add hydrogen atoms when missing from the original structural model. For some structures, disorder of the framework caused hydrogen atoms to be added incorrectly; this issue is resolved by updating the script to ensure the hydrogen addition step only occurs when the polymeric component is identified.

Updated function component.is_polymeric

For further information see
https://downloads.ccdc.cam.ac.uk/documentation/API/modules/molecule_api.html?highlight=is_polymeric#ccdc.molecule.Molecule.is_polymeric

Different types of disorder

Crystallographic disorder is flagged in the CSD differently depending upon the exact nature of the disorder present. It is useful to know the differences between them in order to understand what is included in the non-disordered MOF subset and in a 'non-disordered' search:

- *Non-disordered MOF-subset*: when analysing a submitted structure for inclusion into the non-disordered MOF subset, the algorithm looks for disordered atoms (i.e. cases of multi-site disorder) and then searches for the nearest neighbouring non-disordered atom. If this non-disordered atom is part of the framework, the structure is considered as disordered. If it is not, (i.e. near a solvent molecule), it is considered as non-disordered.
- *A 'non-disordered' search*: it is possible to search for 'non-disordered' structures in the MOF subset using the 'no-disorder' filter in ConQuest or in the API. The result will be different from the already available non-disordered MOF subset. The no-disorder filter excludes entries with any kind of disorder present in the CSD 'disorder' field; including that of unmodelled molecules (commonly seen in MOF-like compounds where disordered solvent is treated using the Platon/Squeeze[3] or Olex2/Mask tools[4])
- *Hydrogen atoms disorder* is classified differently from the disorder of other atoms in the CSD. If the only disorder modelled in a structure involves hydrogen, then the CSD entry will not be considered as disordered and will still appear in a search with the no-disorder filter.

S8. References

1. Moghadam, P. Z.; Li, A.; Wiggin, S. B.; Tao, A.; Maloney, A. G. P.; Wood, P. A.; Ward, S. C.; Fairen-Jimenez, D., Development of a Cambridge Structural Database Subset: A Collection of Metal–Organic Frameworks for Past, Present, and Future. *Chemistry of Materials* **2017,** *29* (7), 2618-2625.
2. Willems, T. F.; Rycroft, C. H.; Kazi, M.; Meza, J. C.; Haranczyk, M., Algorithms and tools for high-throughput geometry-based analysis of crystalline porous materials. *Microporous Mesoporous Mater.* **2012,** *149* (1), 134-141.
3. Spek, A., PLATON SQUEEZE: a tool for the calculation of the disordered solvent contribution to the calculated structure factors. *Acta Crystallographica Section C* **2015,** *71* (1), 9-18.
4. Dolomanov, O. V.; Bourhis, L. J.; Gildea, R. J.; Howard, J. A. K.; Puschmann, H., OLEX2: a complete structure solution, refinement and analysis program. *Journal of Applied Crystallography* **2009,** *42* (2), 339-341.
